# The effectiveness of governmental nonpharmaceutical interventions against COVID-19 at controlling seasonal influenza transmission: an ecological study

**DOI:** 10.1186/s12879-022-07317-2

**Published:** 2022-04-04

**Authors:** Zekai Qiu, Zicheng Cao, Min Zou, Kang Tang, Chi Zhang, Jing Tang, Jinfeng Zeng, Yaqi Wang, Qianru Sun, Daoze Wang, Xiangjun Du

**Affiliations:** 1 School of Public Health (Shenzhen), Shenzhen Campus of Sun Yat-Sen University, Shenzhen, 518107 People’s Republic of China; 2 School of Public Health (Shenzhen), Sun Yat-Sen University, Guangzhou, 510275 People’s Republic of China; 3 Key Laboratory of Tropical Disease Control, Ministry of Education, Sun Yat-Sen University, Guangzhou, 510030 People’s Republic of China

**Keywords:** Nonpharmaceutical interventions, Influenza, Global, Machine learning

## Abstract

**Background:**

A range of strict nonpharmaceutical interventions (NPIs) were implemented in many countries to combat the coronavirus 2019 (COVID-19) pandemic. These NPIs may also be effective at controlling seasonal influenza virus infections, as influenza viruses have the same transmission path as severe acute respiratory syndrome coronavirus 2. The aim of this study was to evaluate the effects of different NPIs on the control of seasonal influenza.

**Methods:**

Data for 14 NPIs implemented in 33 countries and the corresponding influenza virological surveillance data were collected. The influenza suppression index was calculated as the difference between the influenza positivity rate during its period of decline from 2019 to 2020 and during the influenza epidemic seasons in the previous 9 years. A machine learning model was developed using an extreme gradient boosting tree regressor to fit the NPI and influenza suppression index data. The SHapley Additive exPlanations tool was used to characterize the NPIs that suppressed the transmission of influenza.

**Results:**

Of all NPIs tested, gathering limitations had the greatest contribution (37.60%) to suppressing influenza transmission during the 2019–2020 influenza season. The three most effective NPIs were gathering limitations, international travel restrictions, and school closures. For these three NPIs, their intensity threshold required to generate an effect were restrictions on the size of gatherings less than 1000 people, ban of travel to all regions or total border closures, and closing only some categories of schools, respectively. There was a strong positive interaction effect between mask-wearing requirements and gathering limitations, whereas merely implementing a mask-wearing requirement, and not other NPIs, diluted the effectiveness of mask-wearing requirements at suppressing influenza transmission.

**Conclusions:**

Gathering limitations, ban of travel to all regions or total border closures, and closing some levels of schools were found to be the most effective NPIs at suppressing influenza transmission. It is recommended that the mask-wearing requirement be combined with gathering limitations and other NPIs. Our findings could facilitate the precise control of future influenza epidemics and other potential pandemics.

**Supplementary Information:**

The online version contains supplementary material available at 10.1186/s12879-022-07317-2.

## Introduction

Influenza viruses are highly infectious, and they cause seasonal epidemics, leading to 3–5 million severe illness cases [1] and 290,000–650,000 deaths [2] globally each year. Some influenza variants, such as the 1918 and 2009 H1N1 variants, may even cause global pandemics [3, 4]. The next influenza pandemic may occur anywhere and anytime and would cause a major disease burden and result in enormous social and economic costs. Governments should also be well prepared for a rebound of seasonal influenza cases following the relaxation of control measures set in place to contain the coronavirus 2019 (COVID-19) pandemic.

To curb the global COVID-19 pandemic in 2020, governments around the world enforced a range of rigorous nonpharmaceutical interventions (NPIs), such as closing schools and workplaces, ordering mask wearing, and restricting non-essential travel [5]. These NPIs controlled the transmission of severe acute respiratory syndrome coronavirus 2 (SARS-CoV-2), mainly by cutting off its airborne transmission route, a route that is not specific to a single pathogen. Hence, it is reasonable to theorize that these measures would also be effective at suppressing the seasonal transmission of influenza viruses, which have the same transmission path as SARS-CoV-2 [6].

Studies from mainland China [7, 8], Hong Kong [6], Taiwan [9], and Singapore [10] have found that the number of influenza cases in 2020 decreased compared with the number of cases in previous influenza seasons, presumably due to the effectiveness of NPIs at suppressing influenza transmission. However, the scope of these studies was restricted to a specific geographical region, thus limiting the type and intensity level of the NPIs studied. Furthermore, these studies only evaluated the overall effect of a set of NPIs combined, instead of disentangling the specific effects of individual NPIs. The effect of NPIs at different intensity levels also remains unstudied. However, policymakers require more detailed information to facilitate better decision-making and the design of more precise strategies for the effective control of influenza. Machine learning and explainable artificial intellectual methods may effectively fulfill the function of capturing the complex relationship between the presence or intensity of an NPI and the incidence of influenza. However, these powerful methods were not well used in previous studies.

The aim of this study was to quantify and compare the effectiveness of 14 NPIs at suppressing influenza transmission in 33 countries; to identify the optimal NPIs, individually and in combination; and to provide detailed scientific evidence for the design of precise strategies to prevent and control the spread of influenza.

## Methods

### Data sources

NPI data were retrieved from the Oxford Covid-19 Government Response Tracker (OxCGRT) [11]. This dataset provides the level and scope of daily NPIs implemented in almost all countries. There were 19 NPIs included in the database (Additional file 1: Table S1) in the following three broad categories: containment and closure policies (school and workplace closures, public event cancellations, gathering limitations, public transport suspension, stay-at-home requirements, and domestic and international travel restrictions), economic policies (unemployment subsidies, debt/contract relief, fiscal measures, and international support), and health system policies (health education promotion, testing policies, contact tracing, emergency investment in healthcare, investment in vaccines, mask-wearing requirements, and vaccination policies). The following five NPIs with low variance (implemented at a frequency less than 10%) were excluded: fiscal measures, international support, emergency investment in healthcare, investment in vaccines, and vaccination policies. As a result, 14 NPIs were included in our analysis.

Given that some countries implemented NPIs nationwide, whereas others may have only implemented the NPIs in local areas, the intensity value of each NPI was calculated to account for its geographical implementation scope. The intensity value of each NPI was calculated according to the formula provided by OxCGRT (sub-index) [11], which takes the implementation level and implementation scope into account. The details of the 14 NPIs and their various intensity levels are shown in Additional file 1: Table S1.

Global influenza virological surveillance data at the national level were obtained from the World Health Organization (https://www.who.int/influenza/gisrs_laboratory/flunet/en/). These data were mainly collected by the Global Influenza Surveillance and Response System (GISRS), which provides weekly updated information on specimens collected from the respiratory tract, including the collection time and the source of the specimens, the number of specimens received/collected, the number of specimens processed, the number of specimens that tested positive for the influenza virus, the number of influenza A and B virus subtypes detected, and the activity of influenza-like illness. The influenza positivity rate was calculated by dividing the number of specimens that tested positive for the influenza virus by the number of specimens processed.

Given that the influenza epidemic in the Southern Hemisphere in 2020 was almost completely suppressed due to the implementation of NPIs [12], data were only collected from countries in the Northern Hemisphere. However, the quality of data from countries in the Northern Hemisphere varied. To control the data quality, countries with no influenza surveillance data when the NPIs were in effect, and those in which the influenza epidemic did not exhibit a typical seasonal epidemic curve were excluded. The phases of the annual influenza epidemic season (week 40 of 1 year to week 20 of the following year) [13, 14] were extracted, with two researchers (Zekai Qiu and Zicheng Cao) counting the number of epidemic peaks. A peak was defined as going from 0 cases to a number of cases significantly higher than the number at previous time points and then back to 0 cases after at least 1 month. Additionally, the Republic of Korea and Turkey were excluded because the decline curve was too short (less than 1 month) in 2020. If the average number of peaks counted by both researchers was greater than nine, the country was included in the analysis. The Spearman’s correlation coefficient between the two researchers’ peak counts was 0.96 (*p* < 0.001), indicating relatively good agreement. The countries included in the analysis are presented in Additional file 1: Fig. S1 and were spread across three continents (Asia, Europe, and North America). The epidemic curves for the selected and excluded countries are shown in Additional file 1: Figs. S1 and S2. Thirty-three countries were included in the analysis (Additional file 1: Fig. S3).

The effectiveness of the NPIs at suppressing influenza was evaluated based on the influenza season of 2019–2020. During this season, no NPIs implemented at the early stage when the number of cases was increasing, but they were implemented during the later stage of the season when the number of cases had begun declining. To compare the influenza epidemic level during periods with and without NPIs, the weekly influenza positivity rate was obtained for the declining stage each year from 2011 to 2019, when NPIs were absent. The overall influenza positivity rate remained at the same level each year (Additional file 1: Fig. S1). In other word, it showed no upward or downward pattern in each year from 2011 to 2019. Therefore, the influenza positivity rate in 2019–2020 season could be compared with the rates from 2011 to 2019. In addition, the overall influenza positivity rate remained at the same level each year. In other word, it showed no upward or downward pattern of influenza positivity rate from 2011 to 2019. Therefore, the influenza positivity rate in 2019–2020 season could be compared with the rates from 2011 to 2019.

For the influenza epidemic season from 2019 to 2020, the interval from the epidemic peak (the maximum influenza positivity rate, corresponding to time t_0_) to the trough (the minimum influenza positivity rate, corresponding to time t_n_) was considered as the declining phase. Each time point (unit: weeks) during the declining phase from 2019 to 2020 was treated separately as t_d_ (t_d_ consisted of t_0_, t_1_, t_2_, …, t_n_). Next, the average value for the influenza positivity rate at t_d_ was calculated based on the values from 2011 to 2019 as reference. Finally, the effectiveness of the NPIs at controlling influenza (i.e., the influenza suppression index) at t_d_ was quantified as the difference between the influenza positivity rates at t_d_ for the 2019–2020 season and the reference positivity rates.

### Data analysis

In general, NPIs do not usually have an immediate effect after they are implemented, but there is a lag effect [5, 15]. In this study, the time needed for each NPI to reach its maximal level of effect was assumed to vary from 1 to 14 days [15]. To determine the minimal time that each NPI needed to generate its maximal effect, lags of 1 to 14 days for each NPI were examined and the Spearman’s rank correlation coefficient between the influenza suppression index and the intensity of each NPI at each lagged day was calculated. For each NPI, the lagged day with the largest correlation coefficient was selected.

A machine learning model, extreme gradient boosting tree (XGBoost) [16], was used to estimate the individual effectiveness of each NPI at suppressing influenza transmission. Specifically, the dataset was randomly split into a training set and a separate testing set, at a ratio of 8:2. The hyperparameters of XGBoost were optimized using Bayesian optimization, with tenfold cross-validation in the training set to overfit the model. Subsequently, regularization terms (including gamma, alpha, and lambda) from the previous step were increased to reduce model overfitting, which was evaluated in the testing set. The final model was refitted using the entire dataset.

An explainable artificial intelligence algorithm facilitated the interpretation of the machine learning model on the prediction of the outcome (i.e., the influenza suppression index) based on the specific features (i.e., NPIs). SHapley Additive exPlanations (SHAP) [17], which is based on a solid mathematical theory (cooperative game theory) was used to characterize the NPIs that suppressed influenza transmission.

The SHAP tool generated SHAP values, SHAP main effect values, and SHAP interaction values. The contribution of NPIs to the suppression of influenza transmission was derived from the SHAP value; the effectiveness of the intensity level of each NPI and the threshold intensity beyond which the NPIs generated their effects were derived from the SHAP main effect value; and the interaction of each pair of NPIs was derived from the SHAP interaction value. Additionally, the effectiveness of the intensity level of each NPI was classified as “strong,” “moderate,” or “weak” using K-means clustering. Detailed information is provided in Additional file 1.

A range of sensitivity analyses was performed to test the robustness of our contribution results. First, each country was removed one at a time and the analysis was repeated. Second, four countries (Canada, China, Russia, and the United States) with large territories and therefore with high regional heterogeneity were removed from the dataset. Third, the non-normalized influenza positivity rate was used. Fourth, whether the NPIs were implemented nationwide or only within a local area was disregarded by only using the implementation level. Finally, different methods, including (1) a least absolute shrinkage and selection operator (Lasso) with tenfold cross-validation, (2) a random forest algorithm with a hybrid SHAP feature contribution ranking, (3) a random forest algorithm wrapped with sequential feature selection, and (4) a support vector machine wrapped with sequential feature selection, were used to select features.

## Results

### Ranking the contribution and effectiveness of the NPIs

**Fig. 1:**
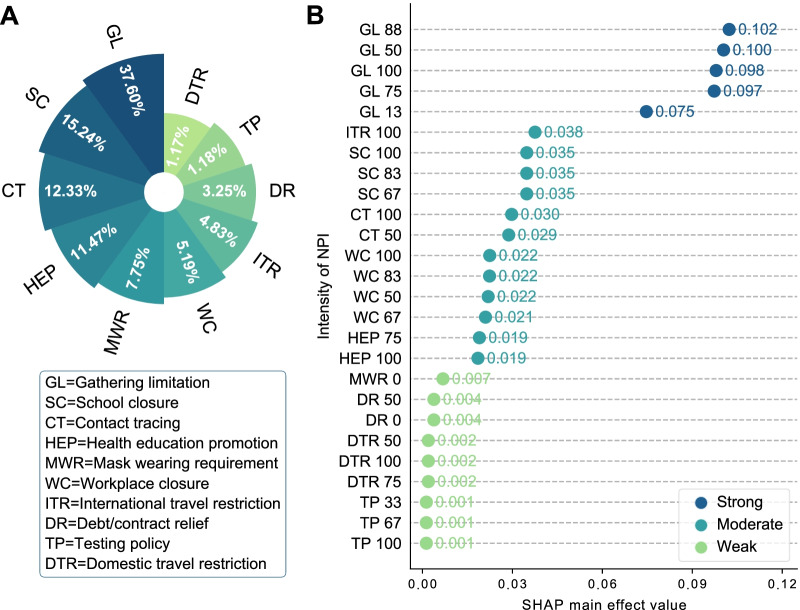
Ranking of the contributions and effectiveness of each nonpharmaceutical intervention (NPI). **A** Percentages of the contribution of the NPIs to suppressing influenza transmission during the declining phase of the 2019–2020 influenza season. **B** The effectiveness of each NPI at different intensity levels. The number after the name of each NPI on the Y-axis represents the intensity level

Four NPIs with negligible effects were excluded by model regularization. Of the remaining 10 NPIs, those with a contribution to the suppression of influenza transmission greater than 10% were gathering limitations (37.60%), school closures (15.24%), contact tracing (12.33%), and health education promotion (11.47%), resulting in a total contribution of 76.64% (Fig. 1A and Additional file 1: Fig. S4). A series of sensitivity analyses consistently confirmed that the finding that these four NPIs made the greatest contribution was robust (Additional file 1: Table S2).

Three groups of NPIs were obtained based on a clustering algorithm, and these groups of NPIs were classified as strong, moderate, or weak based on their effectiveness (Fig. 1B). For gathering limitations, all of the selected intensity levels were classified as strong. The following NPIs were categorized as moderate: (1) banning travel to all foreign regions or total border closure, (2) the closure of some or all schools, (3) limited or comprehensive contact tracing, (4) the closure of some workplaces or all nonessential workplaces, and (5) a coordinated public information campaign. The intensity levels of the remaining NPIs were classified as weak (Fig. 1B).

### The intensity threshold and lag time for the NPIs to have an effect

**Fig. 2:**
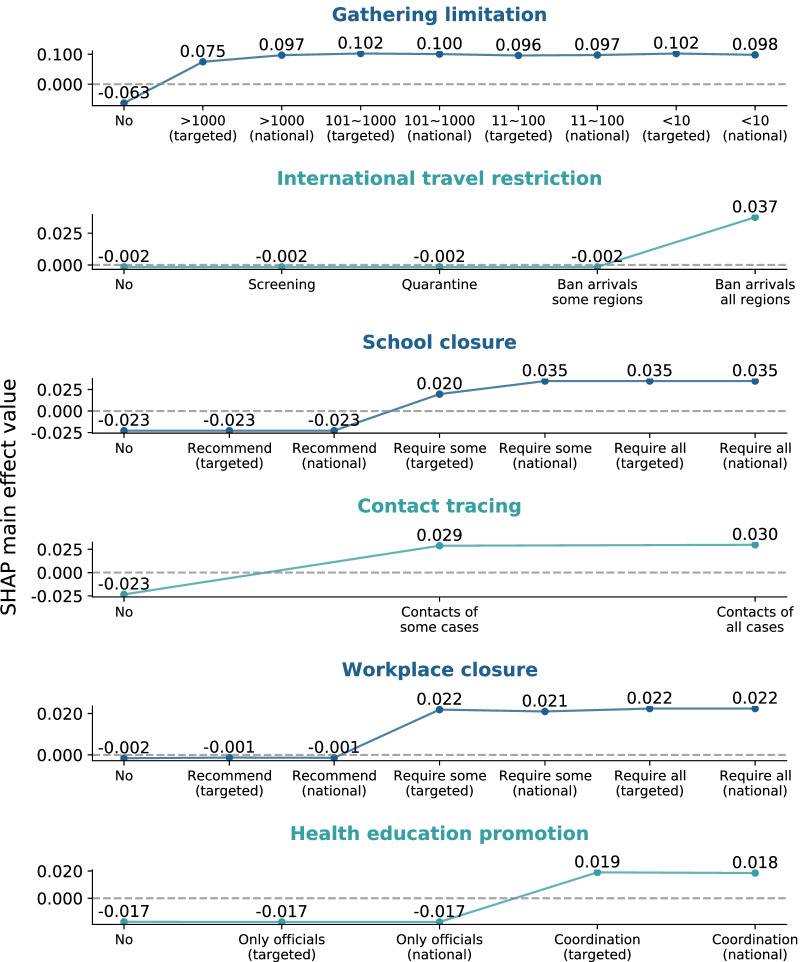
SHapley Additive exPlanations (SHAP) main effect value of each nonpharmaceutical intervention (NPI) at different intensity levels. For each NPI, the first time, its SHAP main effect value exceeded zero indicates the time at which the NPI with the corresponding intensity level started to suppress influenza transmission. At intensities below this level, the NPI did not produce effects. The inflection point in the plot corresponds to the intensity of the NPI that approximately reached the maximal effect

The intensity thresholds of the NPIs with an effectiveness classified as strong or moderate are presented in Fig. 2 (see Additional file 1: Fig. S4 for all 10 NPIs). Restrictions on social gatherings started to have an influenza transmission-suppressing effect when the restrictions were enforced on very large gatherings (> 1000 people), and they reached their maximal effect when expanded nationwide. International travel restrictions started having an effect when travel bans on all regions or total border closures were enforced. School closures started to have an effect when some levels or categories of schools were required to close in specific geographical regions, and they reached their maximum effect when the closures were expanded nationwide. Contact tracing started to have an influenza transmission-suppressing effect when it was performed on a limited number of contacts or performed only for some cases, and it also reached its maximum effect at that intensity level. Workplace closures started to have an effect and also reached their maximal effect when enforced for some sectors or categories of workers (or when employees were required to work from home). Health education promotion started to have an effect and also reached its maximal effect when coordinated public information campaigns (e.g., across traditional and social media platforms) were targeted to a specific geographical region. Increasing the intensity of these aforementioned NPIs beyond the level that generated the maximal effect did not result in obvious improvements (saturation point).

The effects of the introduced NPIs were not immediate. The time needed for each NPI to reach its maximal level of effect varied, but all NPIs reached this level within 1 week (Additional file 1: Fig. S5). Health education promotion required the longest time to have an effect (7 days), followed by school closures and public event cancellation (5 days each). Domestic travel restrictions, international travel restrictions, stay-at-home requirements, public transport suspension, unemployment subsidies, debt/contract relief, and testing policies had lag times of less than 3 days.

### Interactions between pairs of NPIs

**Fig. 3:**
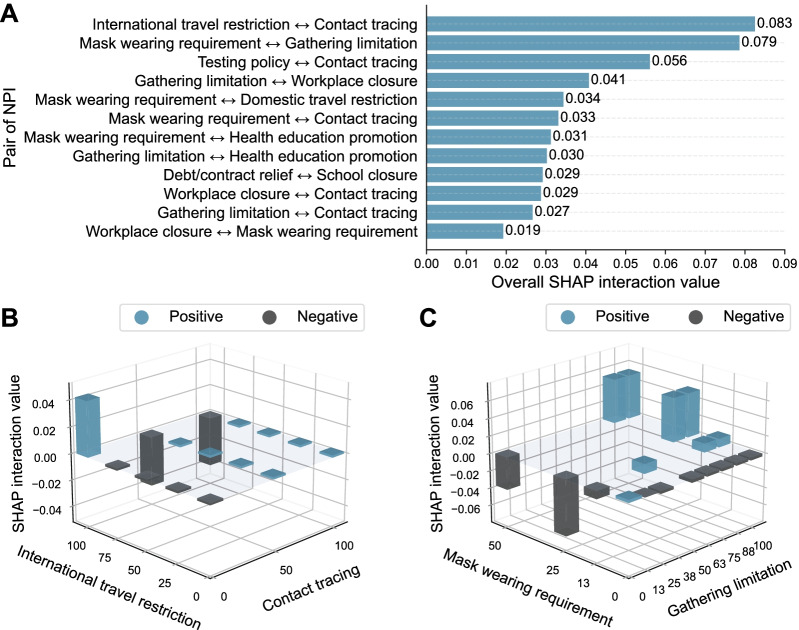
The interaction effects of nonpharmaceutical intervention (NPI) pairs. **A** The overall SHapley Additive exPlanations (SHAP) interaction effect values and the ranking for each NPI pair. **B** The SHAP interaction values for international travel restriction and contact tracing. **C** The SHAP interaction values for mask wearing requirement and gathering limitation. The absence of cubes at the intersection of the X- and Y-axes is due to a lack of data. It does not indicate the lack of an interaction effect

Significant interaction effects between pairs of NPIs were determined (Additional file 1: Figs. S6 and S7). The two pairs of NPIs with the largest overall interaction effects were contact tracing paired with international travel restrictions and mask-wearing requirements paired with social gathering limitations (Fig. 3A). Decreasing the intensity of contact tracing had a positive interaction effect with increasing the intensity of international travel restrictions (Fig. 3B), whereas increasing the intensity of contact tracing had a negative interaction effect with increasing the intensity of international travel restrictions.

Increasing the intensity of the mask-wearing requirement had a positive interaction effect with increasing the intensity of the following NPIs: gathering limitations (Fig. 3C), workplace closures, health education promotion, contact tracing, and domestic travel restrictions (Additional file 1: Fig. S7). However, only increasing the intensity of the mask-wearing requirement, without increasing the intensity of other NPIs, demonstrated a negative interaction effect.

## Discussion

In contrast to the COVID-19 pandemic, influenza epidemics are persistent public health challenges that have occurred many times in human history. However, there is a lack of guidance on formulating NPI strategies to cope with influenza epidemics. To the best of our knowledge, the current study is the first to use a machine learning algorithm combined with an explainable artificial intelligence tool to quantify the effectiveness of COVID-19-targeted NPIs at suppressing influenza transmission. We found that social gathering limitations made the greatest contribution to suppressing influenza transmission. Additionally, we estimated the effectiveness of each intensity level of the NPIs, the intensity and time threshold for each NPI to have an effect, and the interaction between each pair of NPIs.

We found that gathering limitations, school closures, contact tracing, and health education promotion made the greatest contributions to suppressing influenza transmission. These results partially agree with those of previous studies estimating the effectiveness of NPIs against SARS-CoV-2 transmission [18, 19]. We found that school closures were more effective than workplace closures. The reason for this finding may be that the transmission of the influenza virus mainly occurs in children rather than adults. Previous studies have also found that closing schools played an important role in containing the 2009 H1N1 influenza pandemic [20–22].

We found that contact tracing was effective at suppressing influenza transmission, even though this NPI was specific to contact with COVID-19 patients. This may be because the disease symptoms of the two viruses are very similar (e.g., fever and cough), and both viruses are transmitted via the airborne route. Therefore, patients with a history of COVID-19 exposure may also have previously been or are currently exposed to the influenza virus, and patients with suspected COVID-19 symptoms under close tracing and monitoring may actually be infected with the influenza virus. Another reason for this finding may be the high co-infection rate of SARS-CoV-2 and influenza virus. A single-centered retrospective study of 307 COVID-19 patients conducted in Wuhan, China, reported that 57.3% of the patients were also positive for influenza viruses [23]. A recent experimental study found that influenza A virus pre-infection significantly increases susceptibility to SARS-CoV-2 infection [24]. Similarly, other NPIs, such as workplace closures and health education promotion, were also effective at suppressing influenza transmission, regardless of whether the NPI was restricted to regions suspected of having a COVID-19 outbreak or whether the NPI was implemented nationwide or at specific locations. These results may be partly explained by the fact that the two pathogens have the same transmission route, and the results further indicate the existence of interactions between the influenza virus and SARS-CoV-2. Although our results showed that health education promotion had a moderate effect and the longest lag time of the 14 NPIs, its contribution to suppressing influenza transmission in 2020 was sizeable (11.47%). This may be because health education promotion is easy to implement, and it was therefore frequently implemented by many governments globally.

For the first time, we quantified the intensity of each NPI to determine the intensity required to reach its maximum effect. For international travel restrictions, we found that only banning arrivals from certain foreign regions, rather than banning all regions or implementing a total border closure, was not effective at suppressing influenza transmission. The reason for this may be that for viruses capable of causing a global pandemic, such as influenza viruses and SARS-CoV-2, it is crucial to strictly restrict their transmission across international borders. For example, at the early stage of the COVID-19 pandemic, the US only banned travelers from China [25], even when there were emerging cases in Europe. This incomplete restriction of international movement resulted in a surge in COVID-19 cases in the US due to imported cases from Europe [26]. We found that school and workplace closures alone were not sufficient to suppress influenza transmission. This may be because leaders of schools and factories have inadequate knowledge about the threat of infectious diseases and therefore continued normal operation of their organizations. This indicates that governments need to enforce the requirements to close schools and workplaces. For health education promotion, our results showed that an official notice alone was not sufficient to significantly suppress influenza transmission, unless it was combined with a social media campaign. Therefore, the key role of social media in public health education and disease prevention should be recognized, and social media campaigns should be deployed by policymakers. However, false information and rumors spread easily via social media platforms, making the authority of an official notification indispensable.

We found that the NPIs had saturation points. Identifying saturation points may help to maximize the suppression effect, while minimizing the social and economic costs of the interventions. These findings indicate that enforcing NPIs past a certain intensity level may not provide any additional benefits. However, we lacked data regarding the public’s actual behaviors in reaction to the NPIs, which are more relevant to influenza transmission. Therefore, another possible reason for the saturation of the NPIs may be the deterrent effect of a certain intensity level. For example, restricting social gatherings to 1000 participants may also discourage people from attending social activities with less than 1000 participants. However, our analysis was restricted to the earlier stage of the pandemic, and the deterrent effect may decrease during a prolonged pandemic period, because of pandemic-policy fatigue among the public [27].

We took into consideration the lag time of each NPI. The results showed that health education had the longest lag time. This is reasonable, given that it takes a longer time for the public to receive and internalize public health information than information related to other NPIs. We also found that the lag time of some NPIs (e.g., domestic movement restrictions and testing policies) was less than 3 days, which is not reasonable in theory because the series interval (i.e., the time between two successive cases) of influenza is approximately 3 days [28]. This result may be because the effect of these NPIs was too negligible to allow the accurate calculation of the lag time. This is also supported by the low contribution rank of these NPIs.

We found a positive interaction effect when the intensity of the mask-wearing requirement and other NPIs (e.g., gathering limitations) were both high, whereas merely implementing a mask-wearing requirement at a high intensity showed a negative effect. This finding may be explained by risk compensation [29]. In other words, the public may assume that only wearing masks fully protected them from respiratory infections, thereby increasing the frequency and time of contact with others. However, influenza viruses are not only transmitted by air but also by contact (e.g., a person touches a surface with accumulated droplets from an infected person and then touches his/her face) [1]. Therefore, the number of influenza cases may have increased in those people who had close contact with others, despite wearing a mask.

A negative interaction was observed when both the intensity of international travel restrictions and the intensity of contact tracing were high, whereas a positive interaction effect was observed when the intensity of contact tracing was low. This may be because when the intensity of international travel restrictions is high enough to suppress influenza transmission, contact tracing may no longer be necessary due to a decrease in the number of cases. Additionally, during the early declining period of the influenza epidemic, we observed a positive interaction effect when the intensity of both contact tracing and testing policies were high (Additional file 1: Fig. S8C), which may indicate that other NPIs were involved.

Our study has some strengths. First, compared with previous studies that simply used the time of NPI introduction as a surrogate for the effect of the NPI, we used a more reliable, detailed, and comprehensive NPI database, which allowed us to quantify different levels of each NPI. Second, most previous studies [6, 8–10] simply investigated one country or region, whereas we, for the first time, included and compared the effects of various NPIs on influenza transmission across 33 countries in the Northern Hemisphere. Third, previous studies [5, 18, 19] evaluating the effectiveness of NPIs at suppressing COVID-19 spread lacked accurate case data, because at the time, COVID-19 was an emerging infectious disease and detection kits with high sensitivity and specificity were lacking. Importantly, the testing rate is very likely to be influenced by the intensity of testing policies, which vary by country. In comparison, the well-established influenza surveillance system used in this study (i.e., the GISRS) has consistently and reliably monitored influenza activity since 1952 [30]. Finally, we used a machine learning model and an explainable machine learning method to capture the complex relationship between the intensity of the NPIs and the influenza suppression index. Hence, we were able to obtain a range of more detailed and informative results.

Nevertheless, our study has several limitations. First, due to the lack of influenza incidence data in our dataset, we used the influenza positivity rate to approximate actual influenza activity. Although previous studies [31, 32] have used the influenza positivity rate or the multiple influenza-like illness rate to approximate the incidence of influenza, the majority of countries (20/33, 60.6%) in our dataset did not report the influenza-like illness rate. Second, the influenza positivity rate may have been underestimated, because in the context of the COVID-19 pandemic, the health-seeking behavior of influenza patients may have been reduced and medical resources tended to be inadequate. Nevertheless, the number of specimens tested in 2020 was similar to the number tested in the previous year (1,701,758 vs. 1,675,945, respectively) [33]. Third, the 95% confidence intervals of the Spearman’s correlation coefficients of the NPIs with different lag times overlapped (Additional file 2: Table S4). These results may partly be explained by the fact that the lag times of individual NPIs may not be a definite integer number of days, and some NPIs may, in reality, start to have an effect after 84 h (3.5 days). However, since the minimum unit of time for our NPI data was 1 day, it was not possible to specify a smaller unit of time, which may have affected the accuracy of the lag time analysis. In addition, it is possible that the efficiency of different governments at implementing NPIs and the degree of cooperation of the public may have also affected the speed of NPI implementation. Therefore, the number of lag days for the 33 countries included in our analysis may not have been accurate. In summary, we were only able to choose one approximate value that was as close to the true number of lag days as possible. Finally, we used an ecological study design, and therefore, the effectiveness of the NPIs may have been influenced by a range of uncontrolled confounding factors specific to the country in which the NPIs were implemented. These confounding factors may have included the country’s demographic structure and climate and the presence of other NPIs. Due to the same reason, we were unable to adjust for the specific implementation details of each NPI, which may have varied by country. Therefore, the results for interactions between NPIs should be interpreted carefully.

## Conclusions

In conclusion, we estimated the effectiveness of various NPIs at suppressing influenza transmission and provided detailed scientific evidence from different aspects. These results may provide a reference for policymakers to deal with future influenza pandemics. Nevertheless, more detailed information from other aspects, such as other NPIs not included in our analysis and the cost-effectiveness of implementing NPIs, should be explored further.

## Supplementary Information

**Additional file 1. **Additional methods. **Figure S1.** Countries included in the analysis. **Figure S2.** Countries not included in the analysis. **Figure S3.** Countries included in this study. **Figure S4.** SHAP summary plot. **Figure S5.** SHAP main effect value of each NPI at different intensity levels. **Figure S6.** The time need to take effect for each NPI. **Figure S7.** Interaction between each pair of NPIs. **Figure S8.** SHAP interaction value of pair of NPIs. **Figure S9.** Sum of squared errors curves. **Table S1.** The description of each NPI. **Table S2.** Results of sensitivity analysis. **Table S3.** One-sample Wilcoxon test for the SHAP main effect value.

**Additional file 2: Table S4.** Confidence intervals (Median (P0.025, P0.975)) of the spearman correlation coefficients of NPIs with different lag days and the influenza suppression index.

## Declarations

### Availability of data and materials

The policy datasets generated and analyzed during the current study are available in the Oxford COVID-19 Government Response Tracker repository, https://covidtracker.bsg.ox.ac.uk/ [11]. The influenza virological datasets generated and analyzed during the current study are available in the Global Influenza Surveillance and Response System at https://www.who.int/influenza/gisrs_laboratory/flunet/en/. The data and code are available at https://github.com/qqqqqzk/NPI-against-influenza.
