# Supplementary material for: The effectiveness of governmental nonpharmaceutical interventions against COVID-19 at controlling seasonal influenza transmission: an ecological study
Source: BMC Infect Dis. 2022 Apr 4;22:331. doi: 10.1186/s12879-022-07317-2 (PMC8977560; doi:10.1186/s12879-022-07317-2)
Supplement: Supplementary file 1 — Additional file 1. Additional methods. Figure S1. Countries included in the analysis. Figure S2. Countries not included in the analysis. Figure S3. Countries included in this study. Figure S4. SHAP summary plot. Figure S5. SHAP main effect value of each NPI at different intensity levels. Figure S6. The time need to take effect for each NPI. Figure S7. Interaction between each pair of NPIs. Figure S8. SHAP interaction value of pair of NPIs. Figure S9. Sum of squared errors curves. Table S1. The description of each NPI. Table S2. Results of sensitivity analysis. Table S3. One-sample Wilcoxon test for the SHAP main effect value. [file 12879_2022_7317_MOESM1_ESM.docx]

**Table of Contents**

[Additional methods 2](#_Toc95816318)

[Figure S1: Countries included in the analysis. 3](#_Toc95816319)

[Figure S2: Countries not included in the analysis 13](#_Toc95816320)

[Figure S3: Countries included in this study. 27](#_Toc95816321)

[Figure S4 SHAP summary plot: 28](#_Toc95816322)

[Figure S5: SHAP main effect value of each NPI at different intensity levels. 29](#_Toc95816323)

[Figure S6: The time need to take effect for each NPI 32](#_Toc95816324)

[Figure S7: Interaction between each pair of NPIs. 33](#_Toc95816325)

[Figure S8: SHAP interaction value of pair of NPIs 34](#_Toc95816326)

[Figure S9 Sum of squared errors curves 36](#_Toc95816327)

[Table S1 The description of each NPI 38](#_Toc95816328)

[Table S2 Results of sensitivity analysis 42](#_Toc95816329)

[Table S3 One-sample Wilcoxon test for the SHAP main effect value 44](#_Toc95816330)

[References 46](#_Toc95816331)

# Additional methods

1. **Concept list**

- XGBoost(1): a gradient boosting tree based method that builds multiple decision trees step by step and uses a stacking strategy to aggregate these decision trees, achieving state-of-the-art results in many machine learning tasks.
- SHAP(2): a post hoc interpretation algorithm developed for machine learning methods, which is inspired by game theory and has a solid mathematical theoretical foundation with properties including local accuracy, missingness, and consistency.
- Lasso(3): a linear model containing an L1 regular term, which can reduce the coefficients of insignificant variables to 0, thus screening out insignificant variables and reducing the variable dimensionality of the model.
- Random forest(4): a robust predictive model by randomly sampling variables and individuals multiple times, then building multiple decision trees and integrating the results of these decision trees by bagging strategy.
- Support vector machine(5): The support vector machine finds the hyperplane in the high-dimensional space that could distinguish each class, and this hyperplane maximizes the distance of the nearest points of each class.
- Forward Sequential feature selection(6) (SFS) is a greedy algorithm that starts with no variables and gradually adds one variable that maximizes the reduction of the model prediction error.
- Effectiveness: The suppressive effect of various NPIs on the spread of the influenza virus.
- Contribution: The suppressive effect of the NPI on influenza virus transmission during the declining phase of the 2020 influenza epidemic season, taking into account the effectiveness of the NPI and the frequency of NPI implementation.
- Interaction: The positive interaction effect is an additional effect of the combination of two NPIs at specific intensity levels on suppression of influenza transmission, whereas the negative interaction is a reduced effect on suppression of influenza transmission.

1. **Introduction of the SHAP value**

Specifically, each intensity level of an NPI has a SHAP value(2), which was calculated conditioning on all possible NPI orderings. The size of the SHAP value of a NPI at an intensity level represents the change of predicted value caused by this NPI at this intensity level. The model predicted value was calculated using the following formula:

$$y=\sum_{i=1}^{M} {SHAP}_{i}+\phi$$

in which $M$ stands for the number of NPIs, and $\phi$ stands for mean predicted value.

The model predicted value could be approximated to the sum of the SHAP value of each NPI adding the mean predicted value. Each NPI’s contribution in suppressing the influenza transmission at 2019-2020 influenza season was calculated by the sum of positive SHAP values.

The SHAP main effect value of each NPI at each intensity level was calculated by the difference between the SHAP value and the sum of each SHAP interaction value according to the following formula.

$${SHAP}_{i,i}={SHAP}_{i}-\sum_{i\neq j} {SHAP}_{i,j}$$

which $j$ stands for other NPIs interacted with NPI $i$.

The statistical significance of the effectiveness (SHAP main effect value) of each NPI at different intensity levels in suppressing influenza transmission were tested by the one-sided Wilcoxon signed-rank test.(7) The average values of those NPI intensity levels with p < 0.05 were ranked, and then grouped the NPI intensity levels into “strong”, “moderate”, and “weak” by using K-means clustering.

Given that many governments rolled out several NPIs simultaneously, the interaction effect among all pairs of NPIs at each intensity level were assessed using SHAP interaction value. The positive interaction effect is an additional effect of the combination of two NPIs at specific intensity levels on suppression of influenza transmission, whereas the negative interaction is a reduced effect on suppression of influenza transmission. All intensity levels of each NPI were divided into two groups (effective vs ineffective) based on the results of SHAP main effect value. In each of the two groups, the SHAP interaction values at different levels were averaged as group SHAP interaction values. For each pair of NPIs, the overall SHAP interaction value was calculated by taking the sum of the four absolute group SHAP interaction values. The two pairs of NPIs with the largest overall SHAP interaction effect were further explored.

1. **The procedure of verifying assumptions of Wilcoxon test**

3.1 For the first assumption “Dependent samples”, because we used the One-Sample Wilcoxon signed-rank test, where the effect of the NPI intensity for a single NPI was compared to the effect without implementation of that NPI (effect of 0), it is a difference between before and after measurement and the assumption is naturally satisfied.

3.2 For the second assumption “Independence”, the independence between SHAP values was satisfied. Because according to the SHAP approach illustrated in the developer article (A Unified Approach to Interpreting Model Predictions10, Section 2: Additive Feature Attribution Methods: we focus on local methods designed to explain a prediction f(x) based on a single input x, as proposed in LIME), each individual SHAP value was computed for each individual. Therefore, the SHAP value calculation does not depend on other individuals.

3.3 For the third and fourth assumptions “Continuous dependent variable” and “Ordinal level of measurement”: SHAP main effect value is a quantitative indicator instead of a categorical indicator, therefore the Wilcoxon sign test could be used.

1. **The determination of number of initial centroids of k-means cluster**

“elbow method” and “silhouette method” were used to determine the optimal number of centroids for K-means clustering(8, 9). The results of the elbow method and the silhouette method were shown in Figure S9. Choosing the turning point based on those two methods and considering our purpose to divide the NPI effectiveness into three categories of strong, moderate, and weak, the number of initial centroids to 3 was chosen.


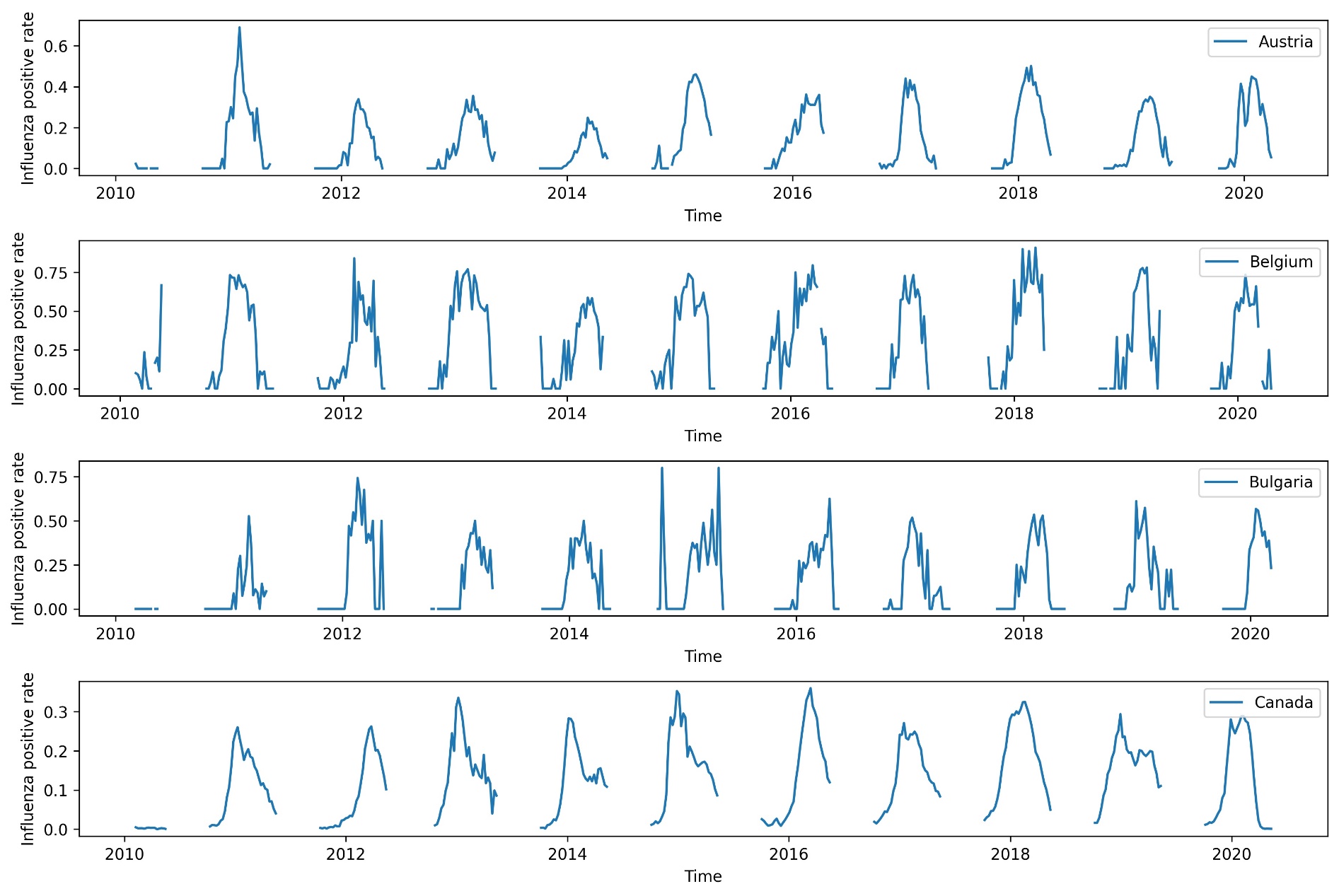

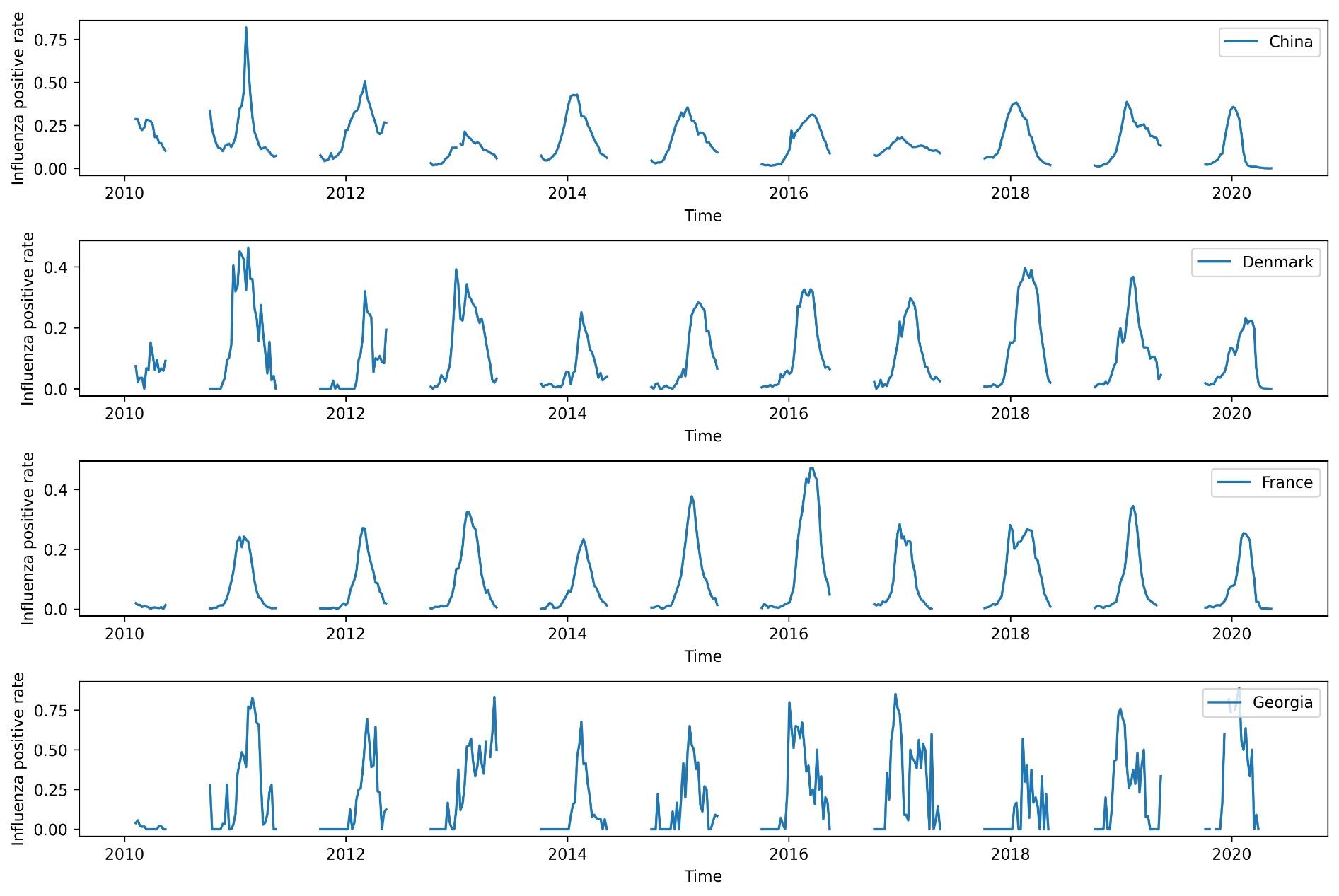

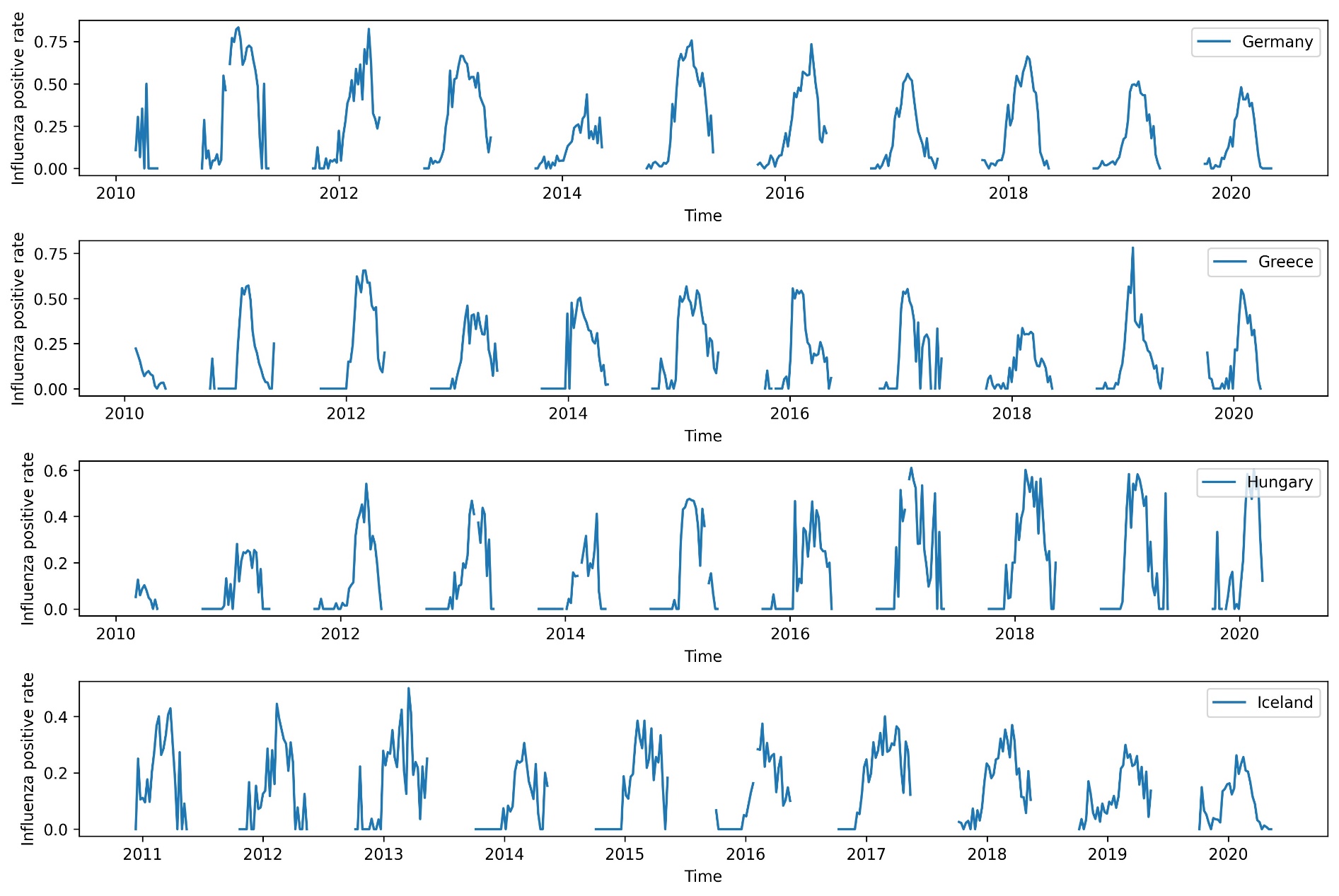

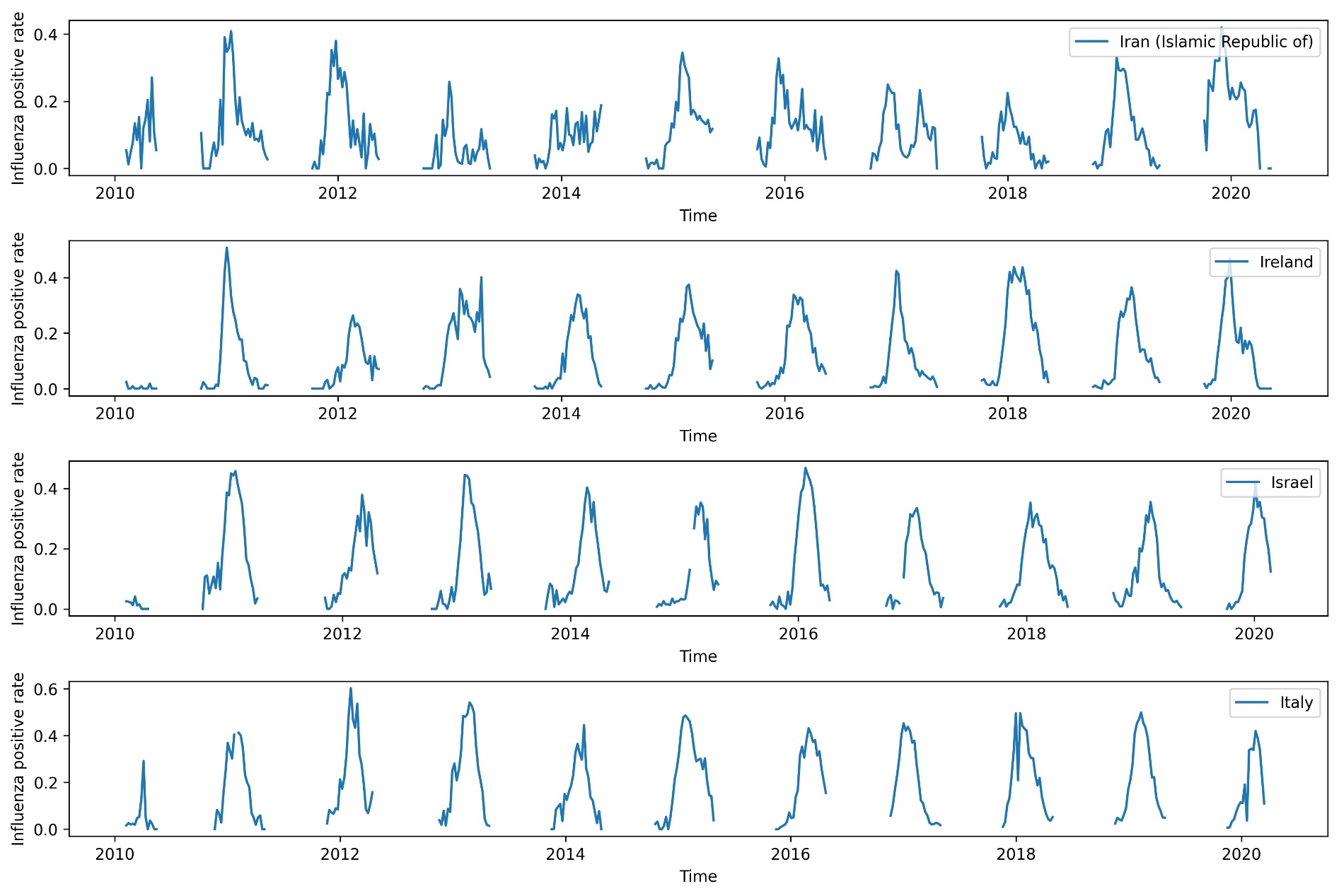

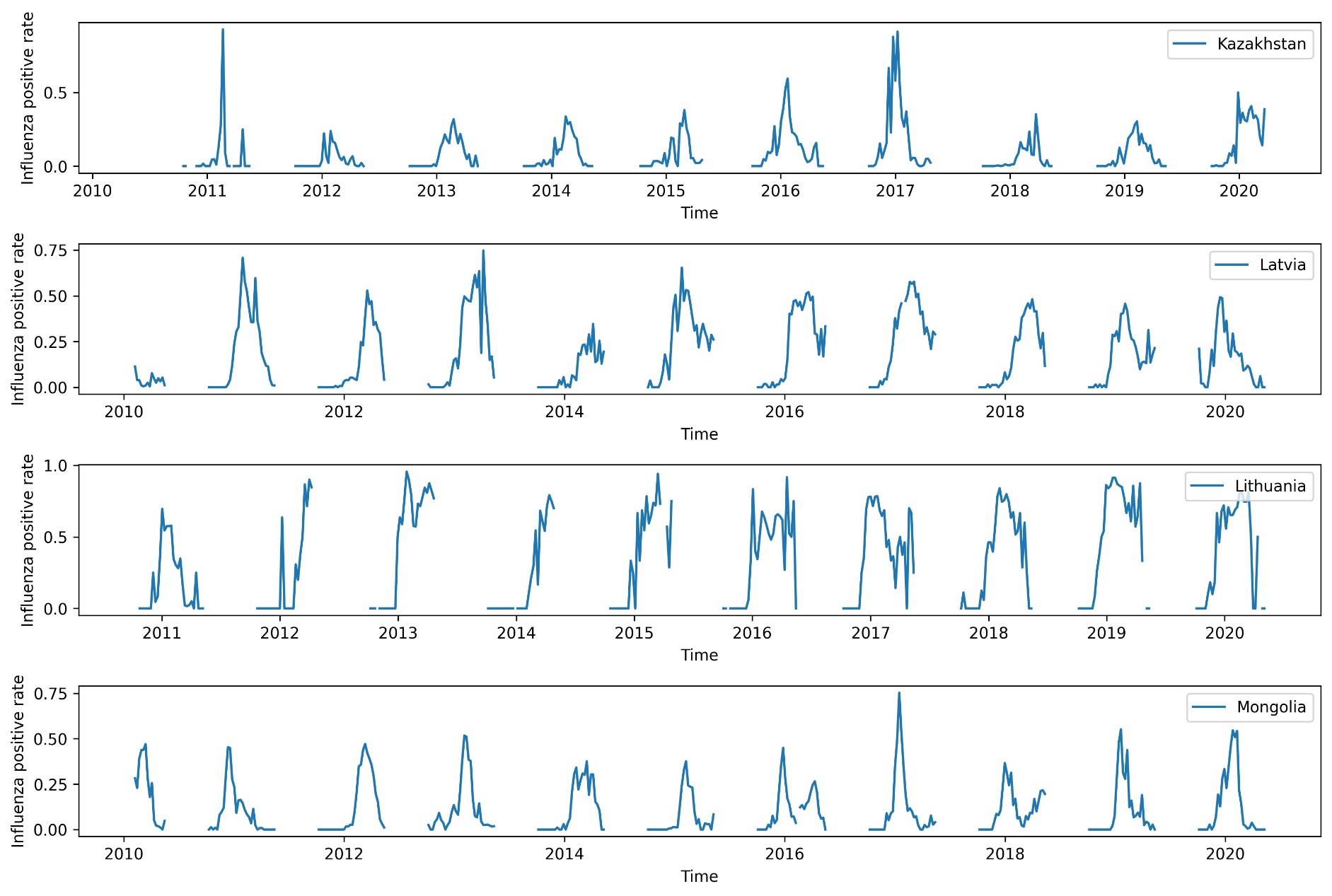

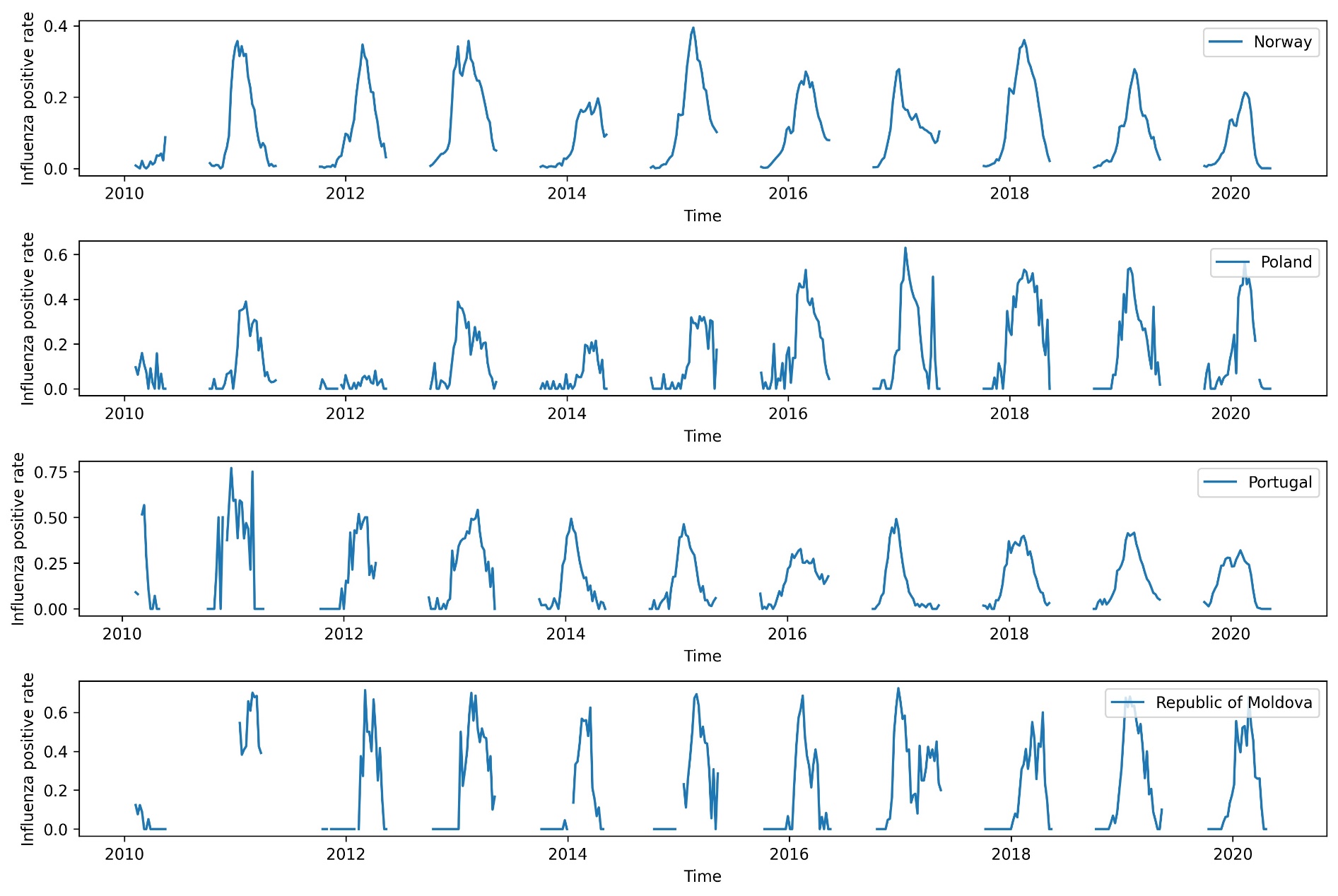

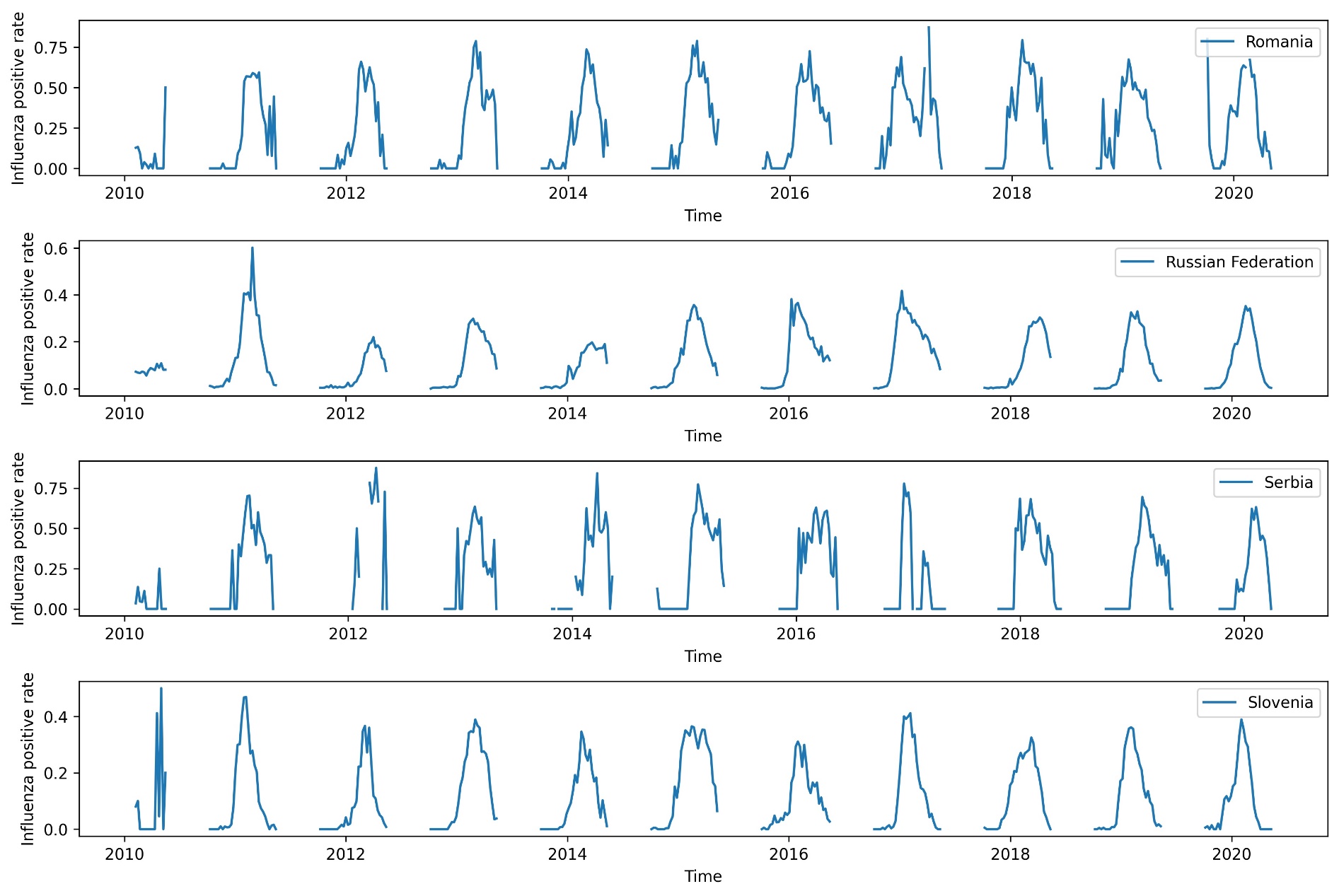

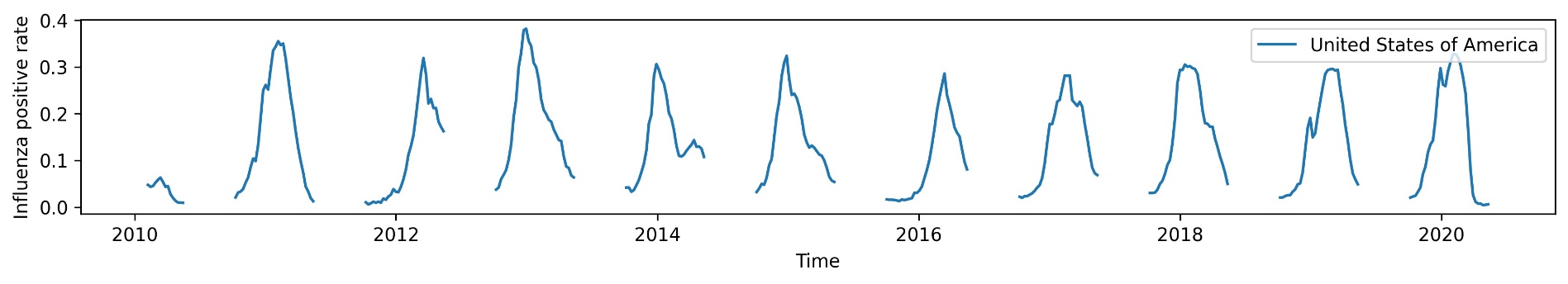


# Figure S1: Countries included in the analysis.


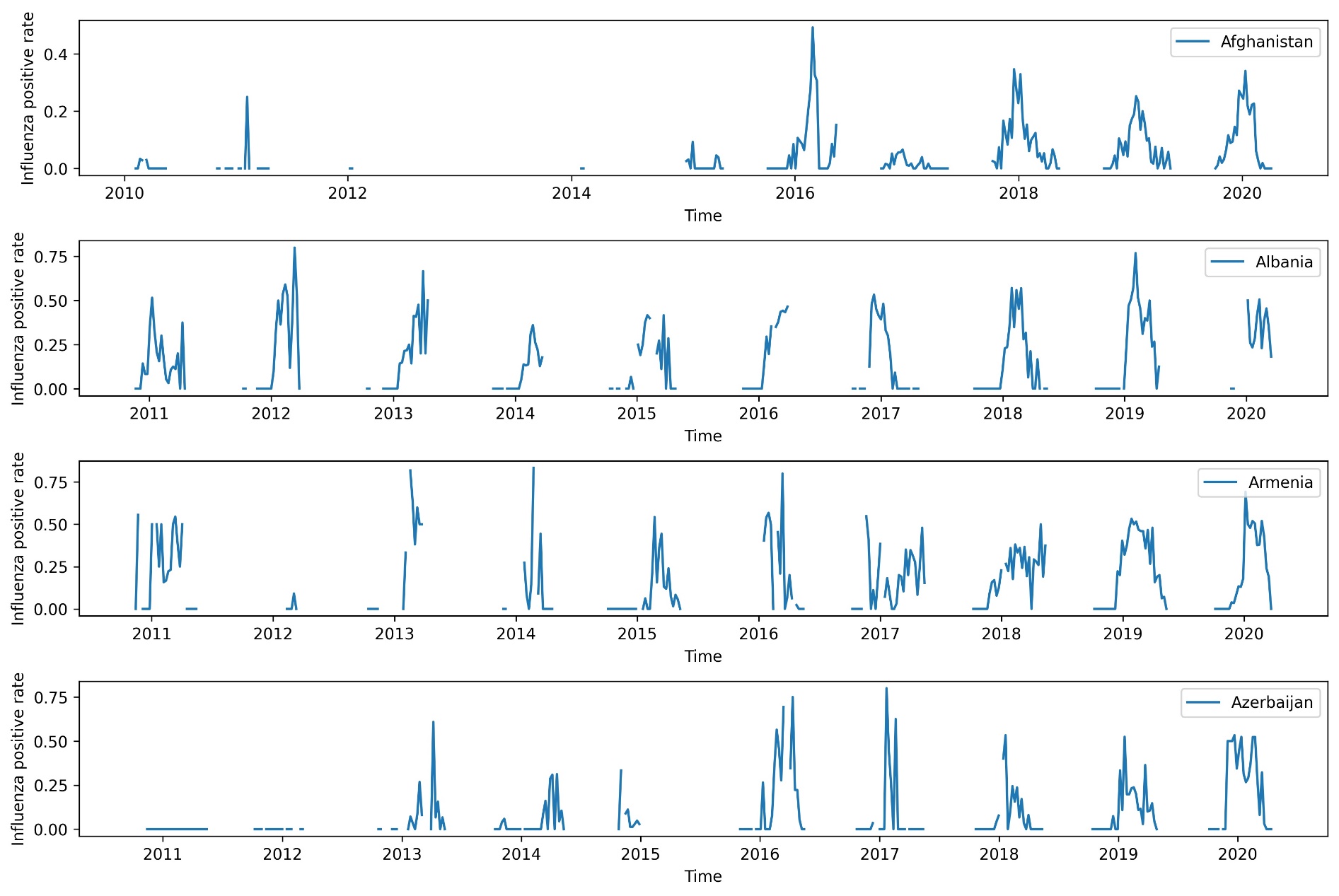

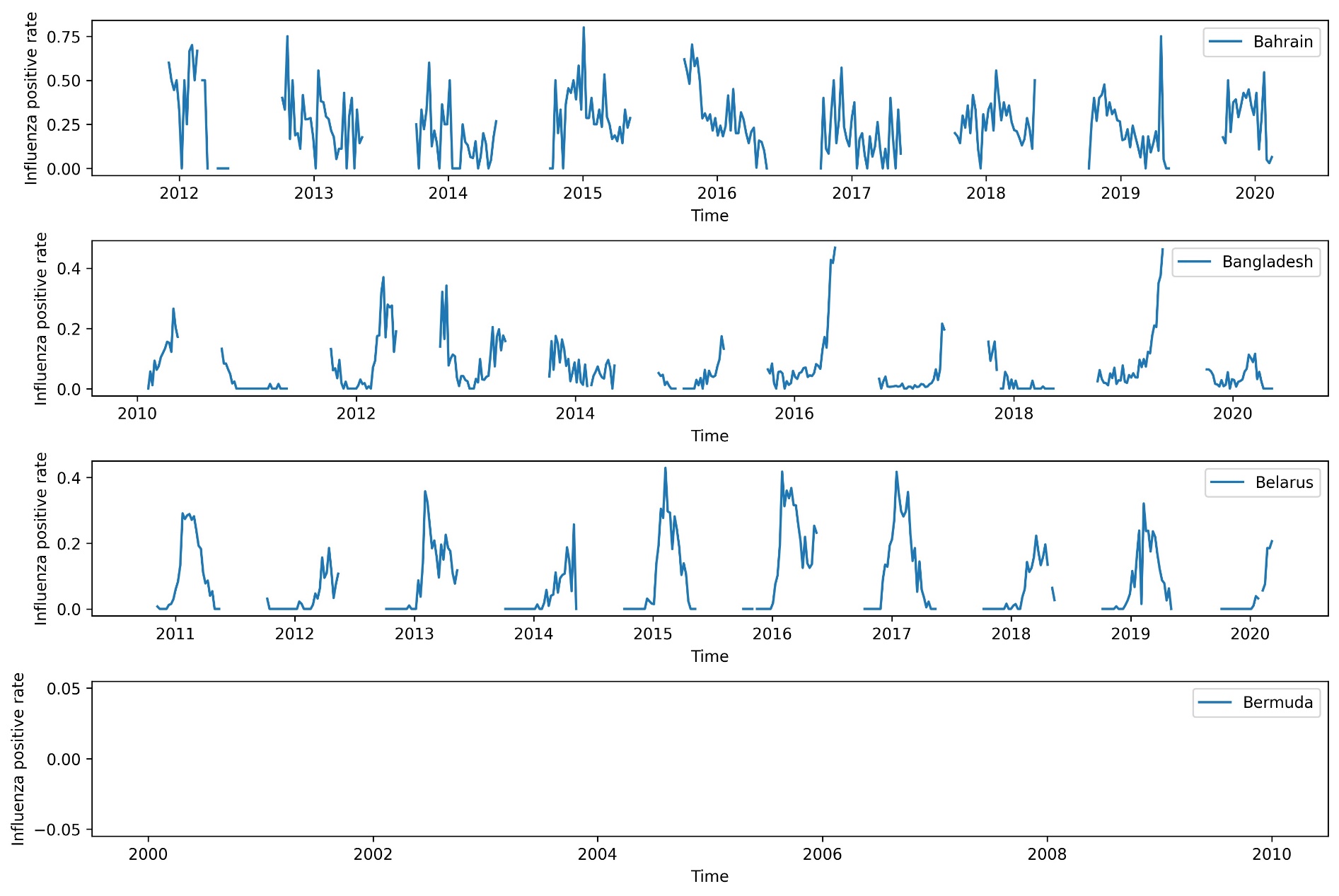

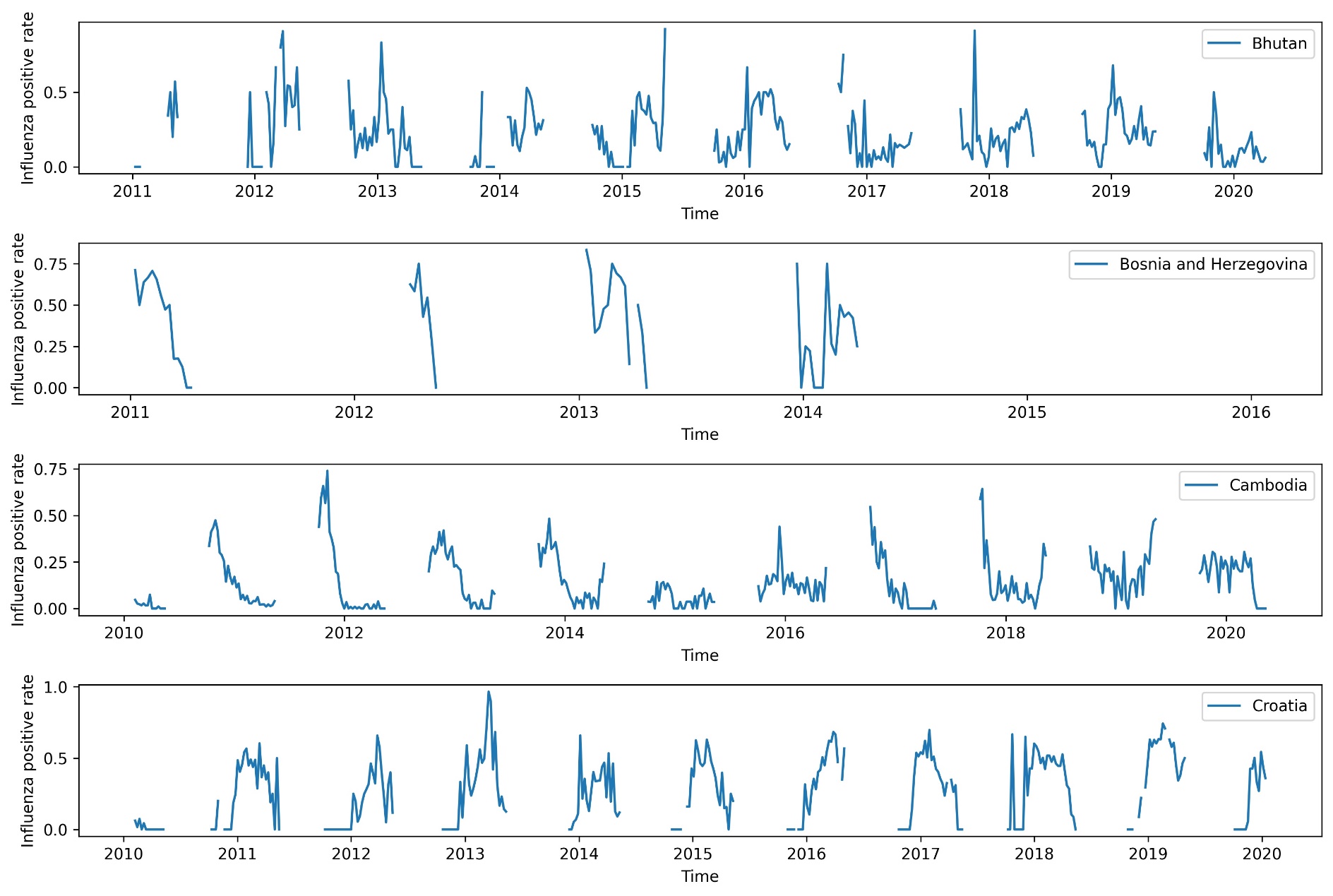

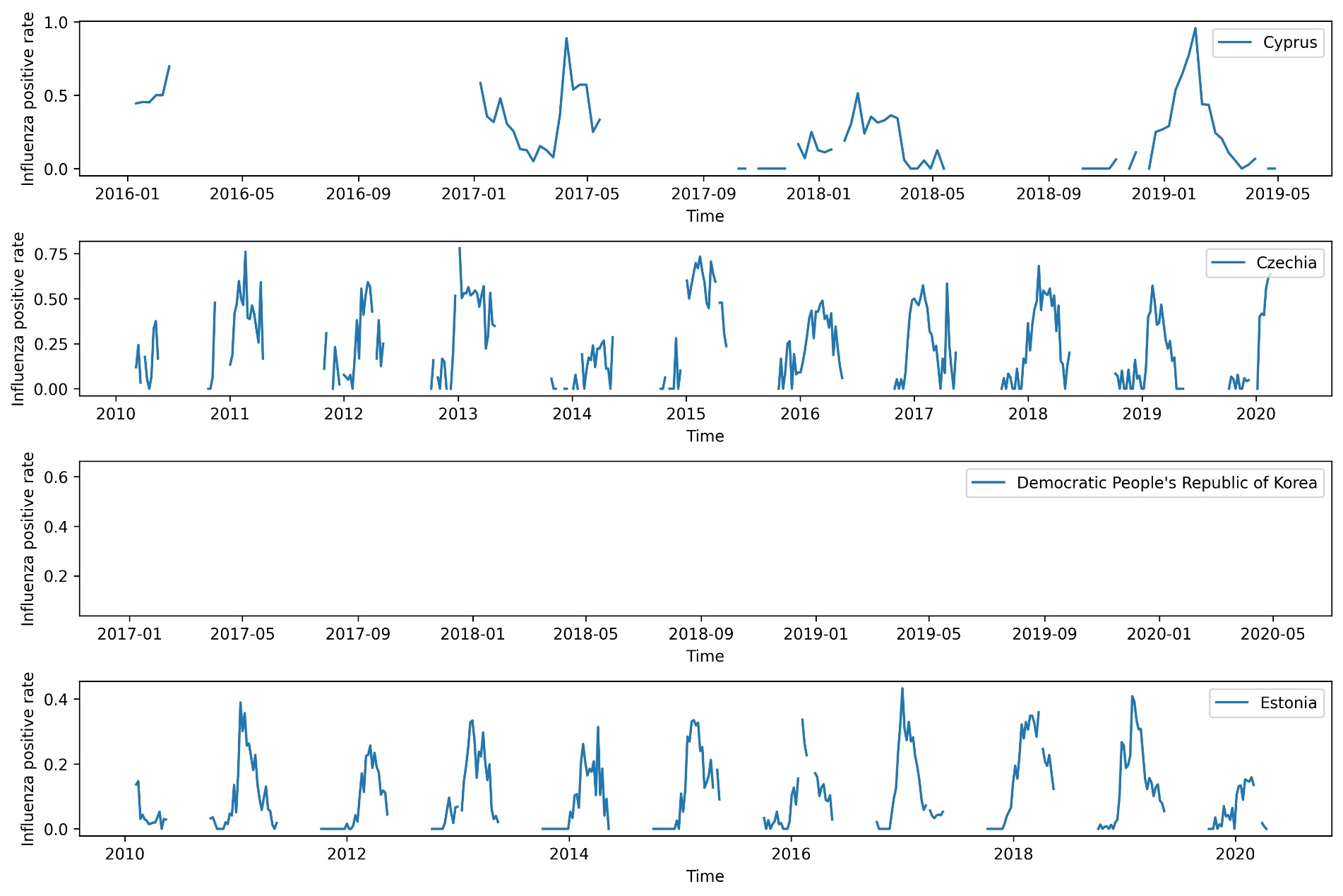

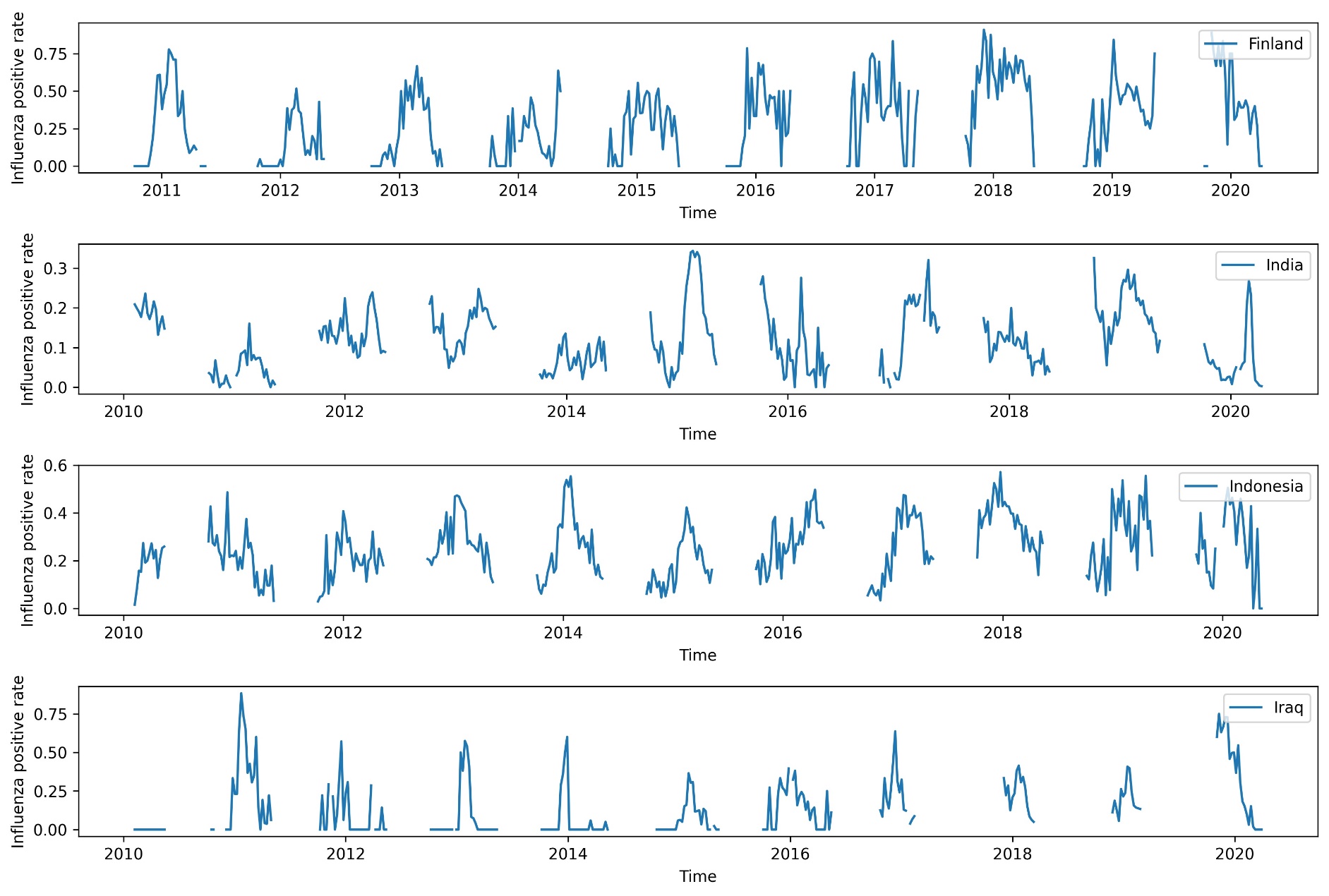

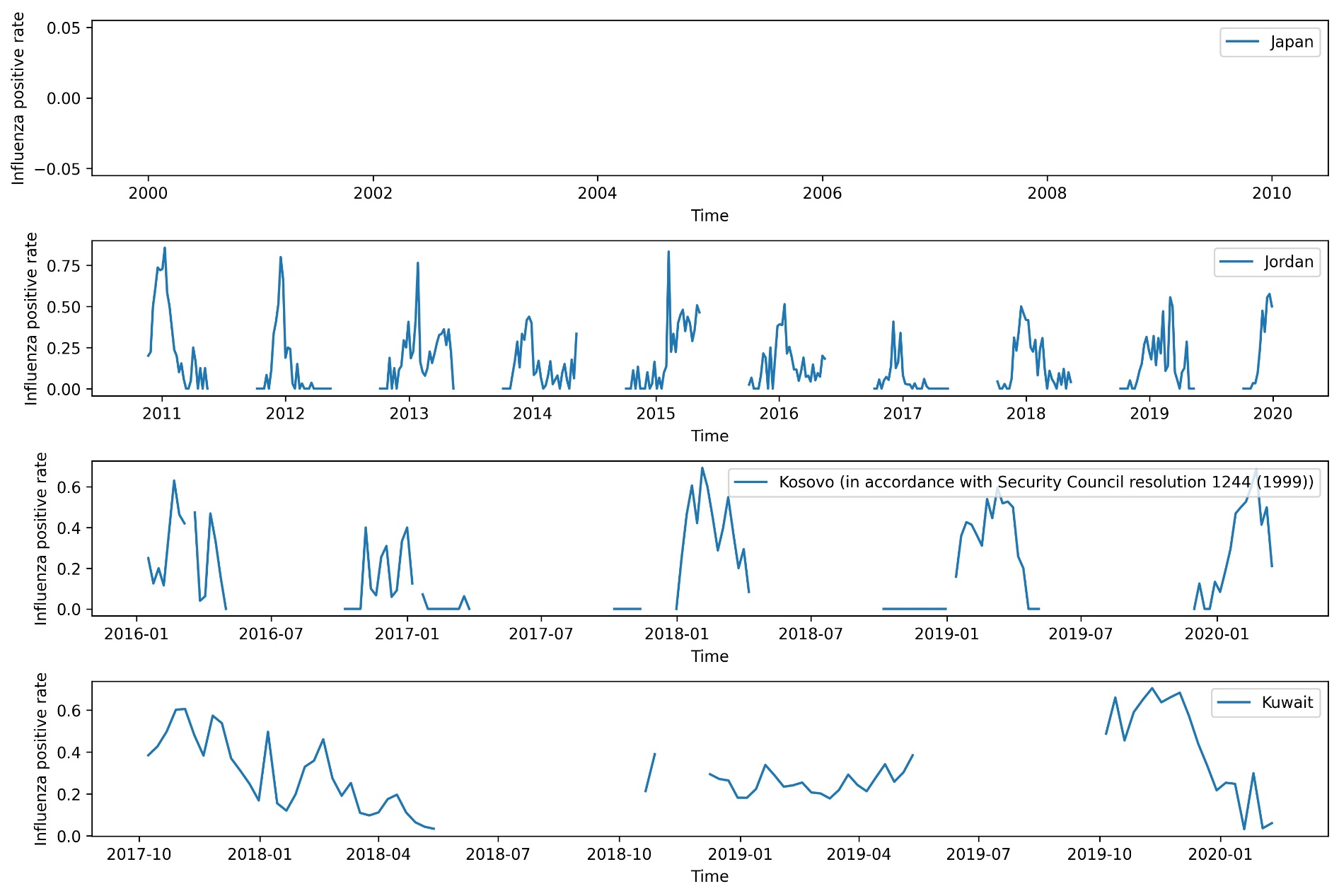

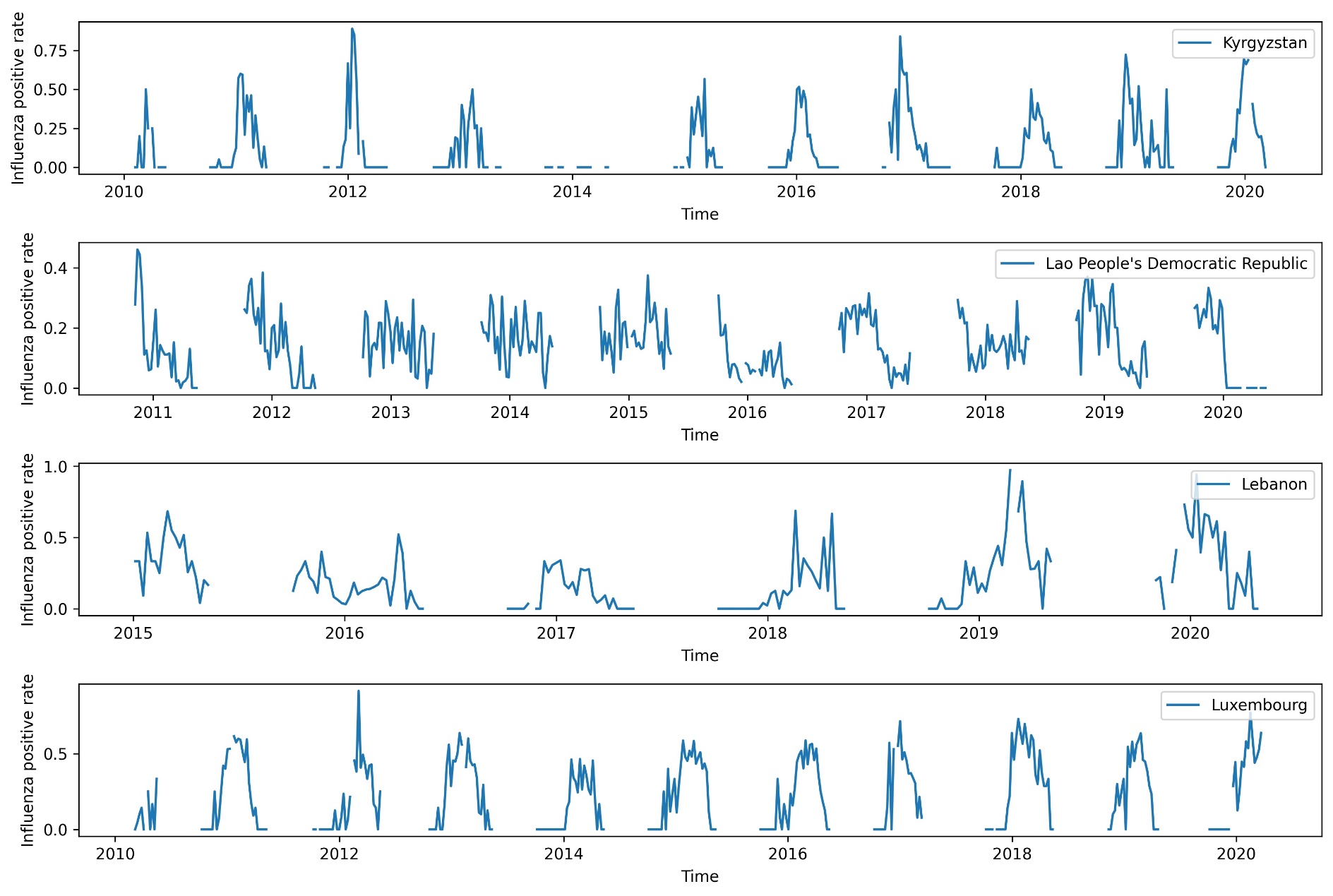

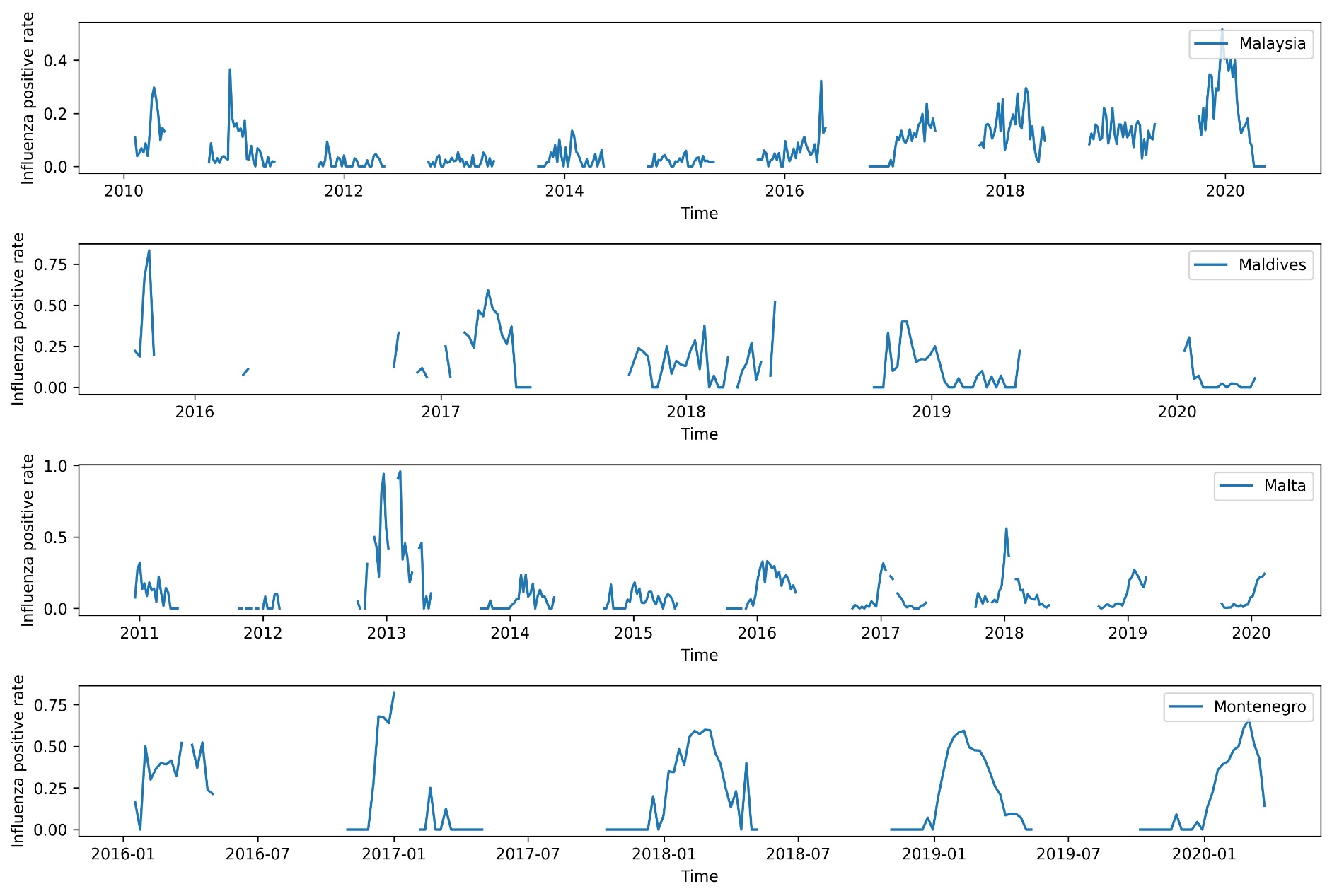

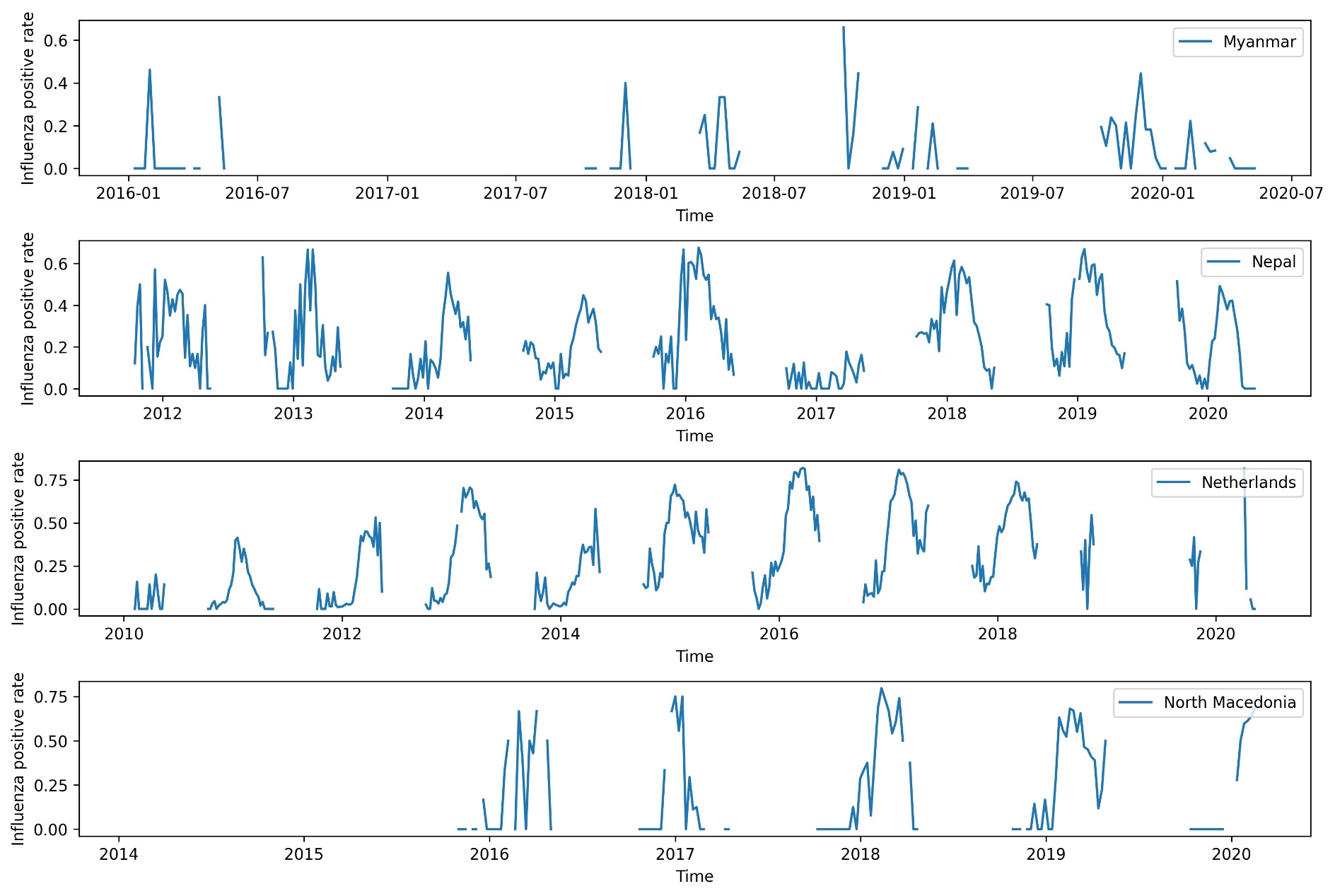

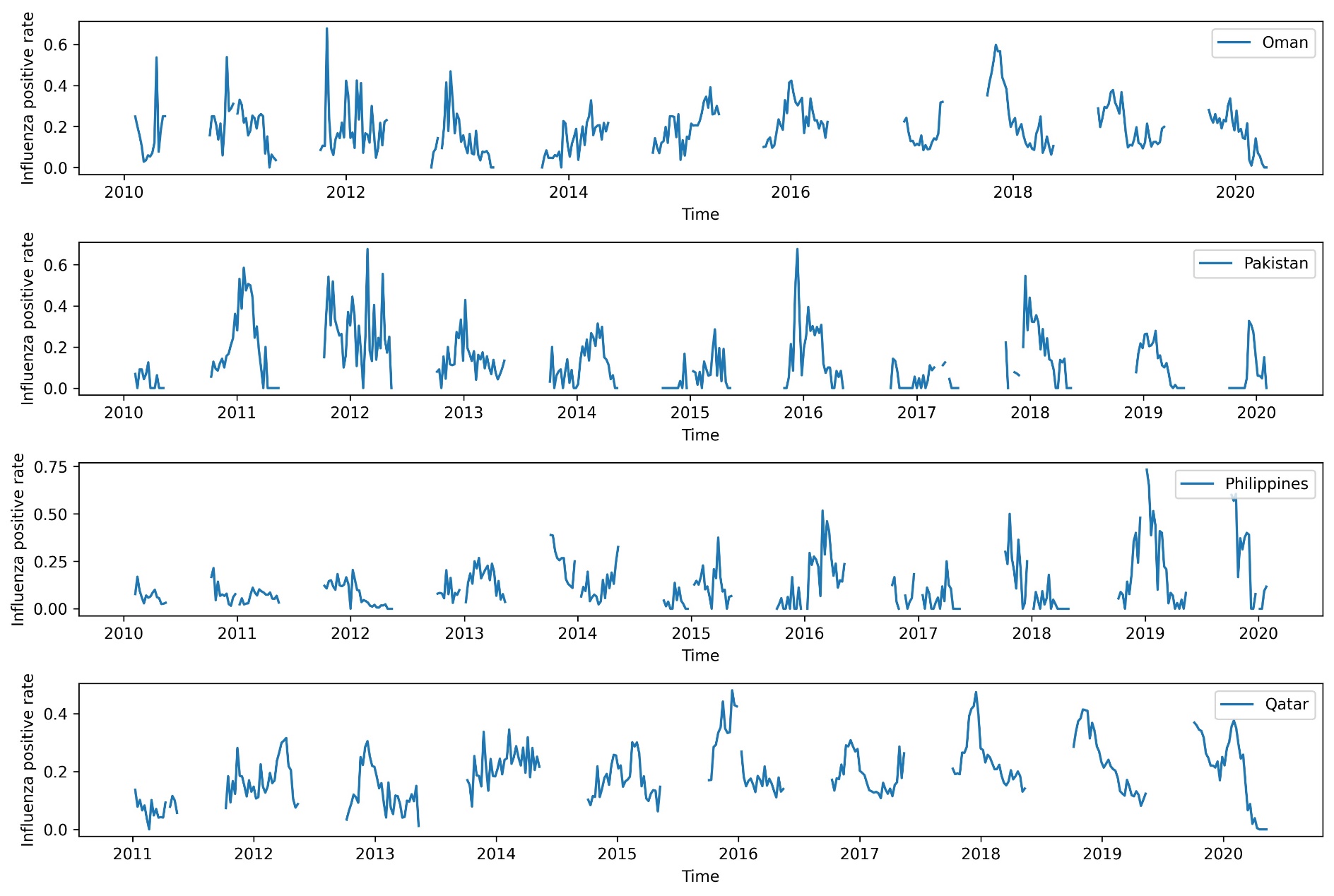

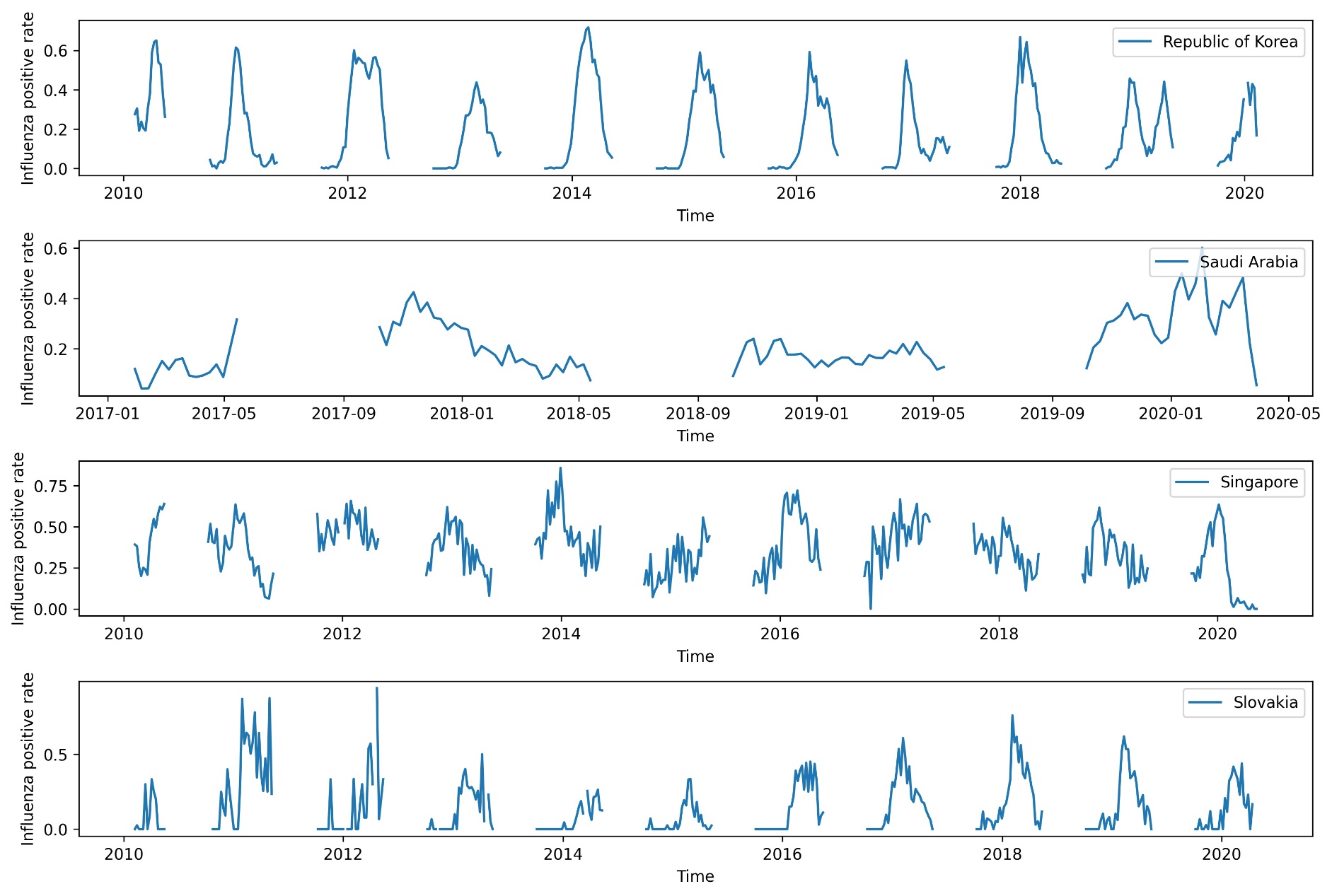

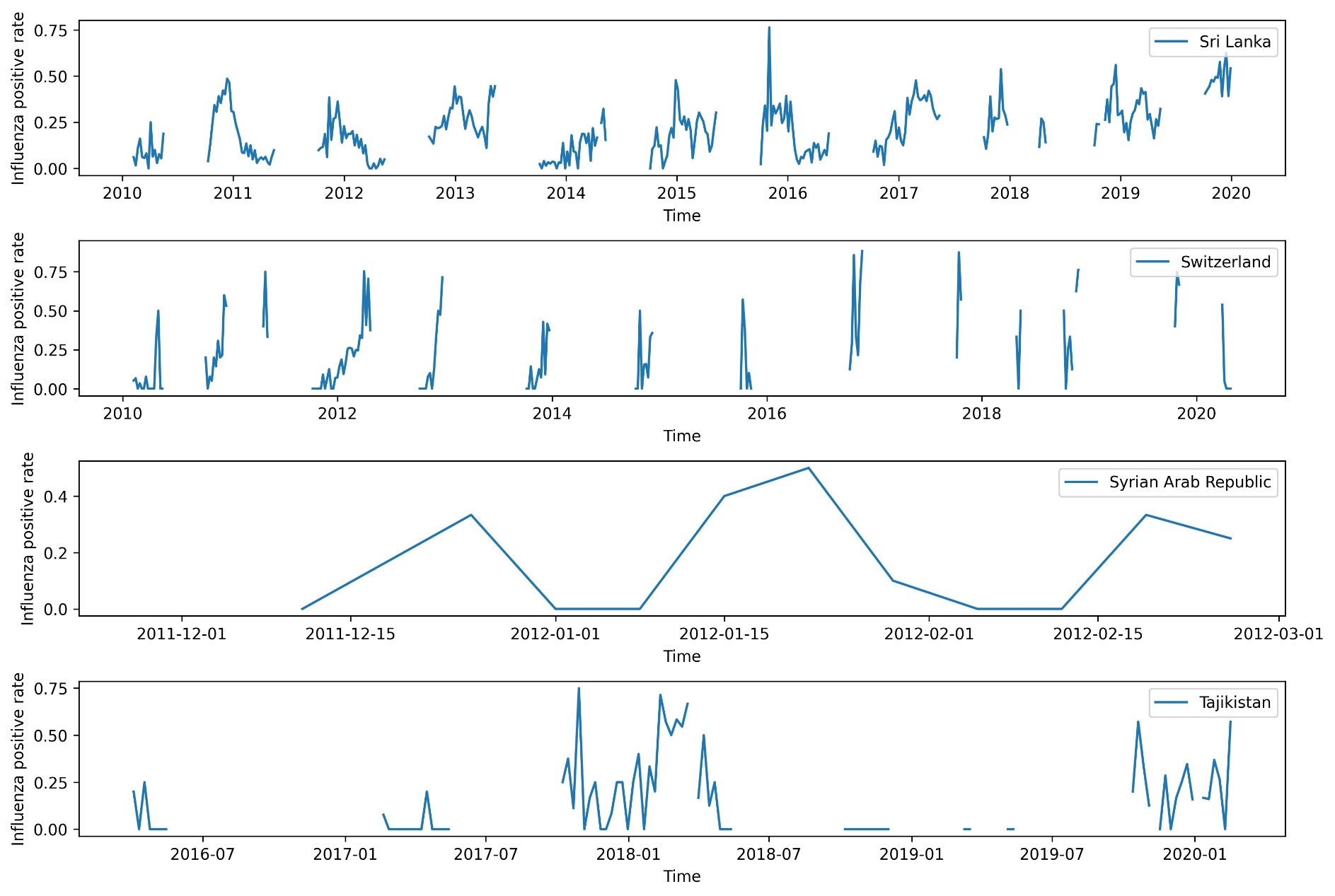

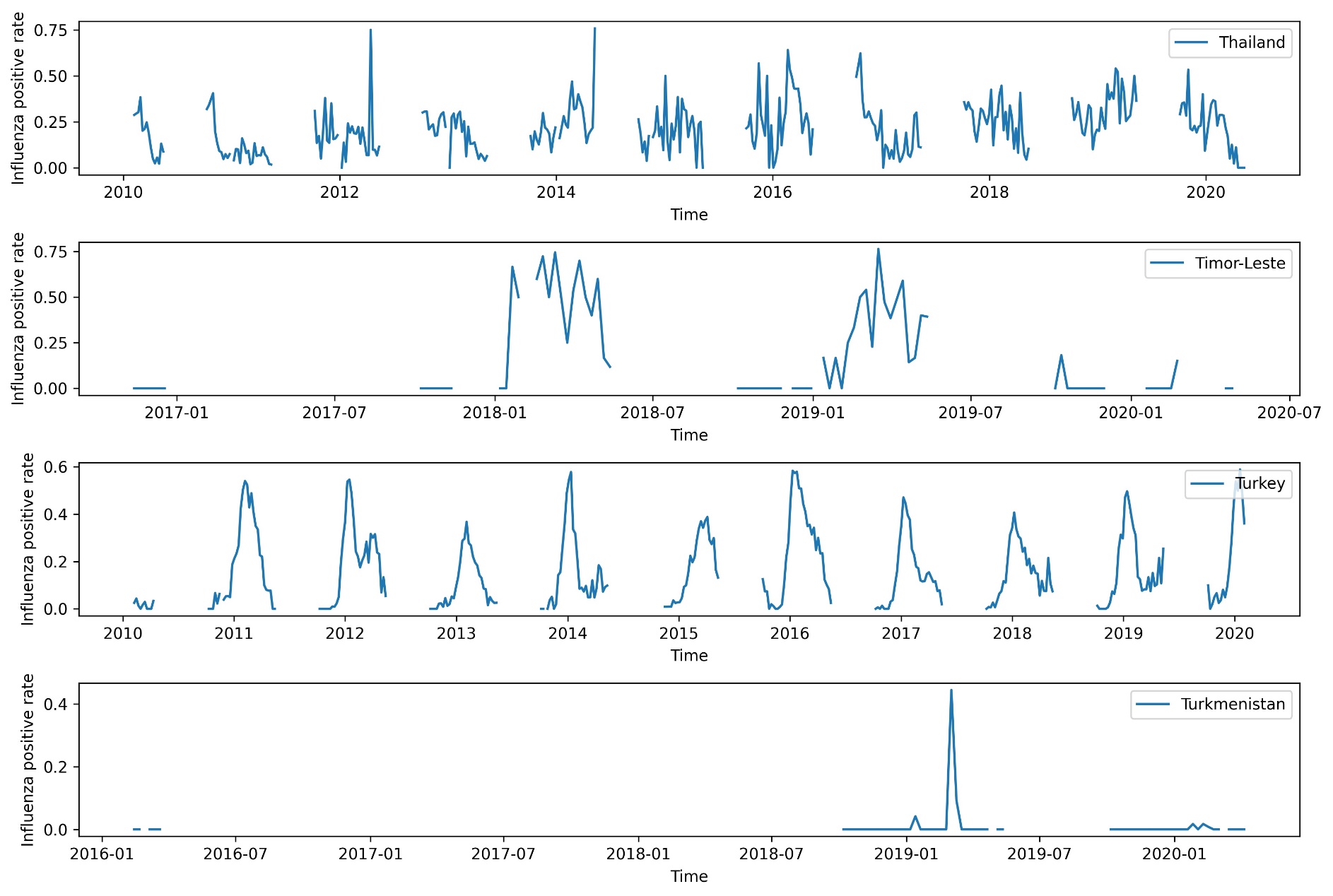

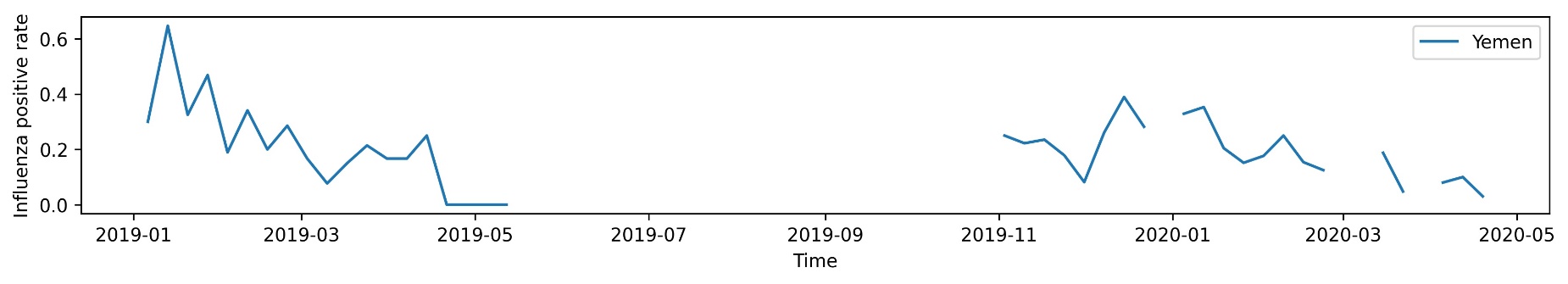


# Figure S2: Countries not included in the analysis


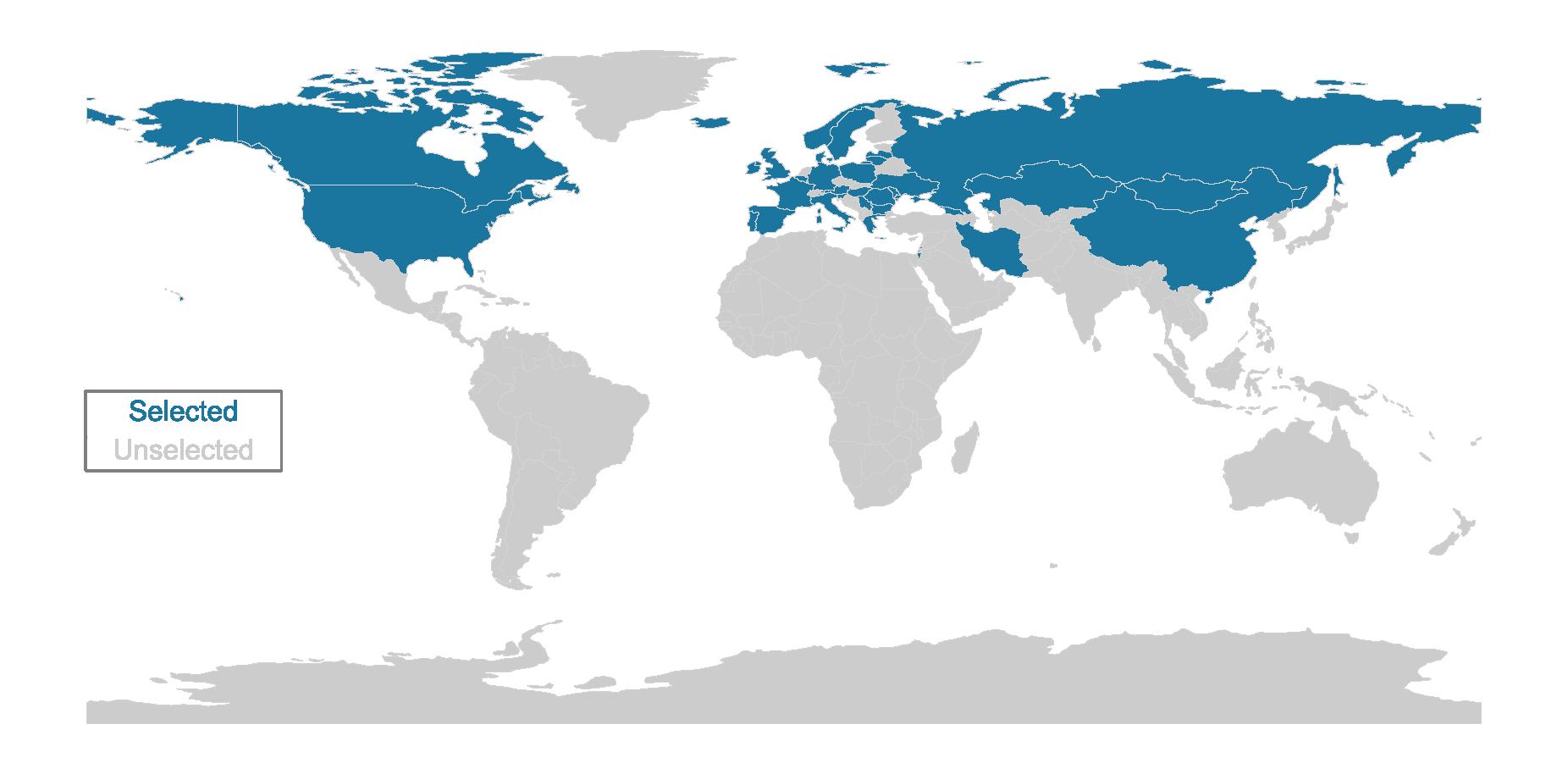


Figure S3: Countries included in this study. (Including Austria, Belgium, Bulgaria, Canada, China, Denmark, France, Georgia, Germany, Greece, Hungary, Iceland, Iran (Islamic Republic of), Ireland, Israel, Italy, Kazakhstan, Latvia, Lithuania, Mongolia, Norway, Poland, Portugal, Republic of Moldova, Romania, Russian Federation, Serbia, Slovenia, Spain, Sweden, Ukraine, United Kingdom of Great Britain and Northern Ireland, and United States of America)


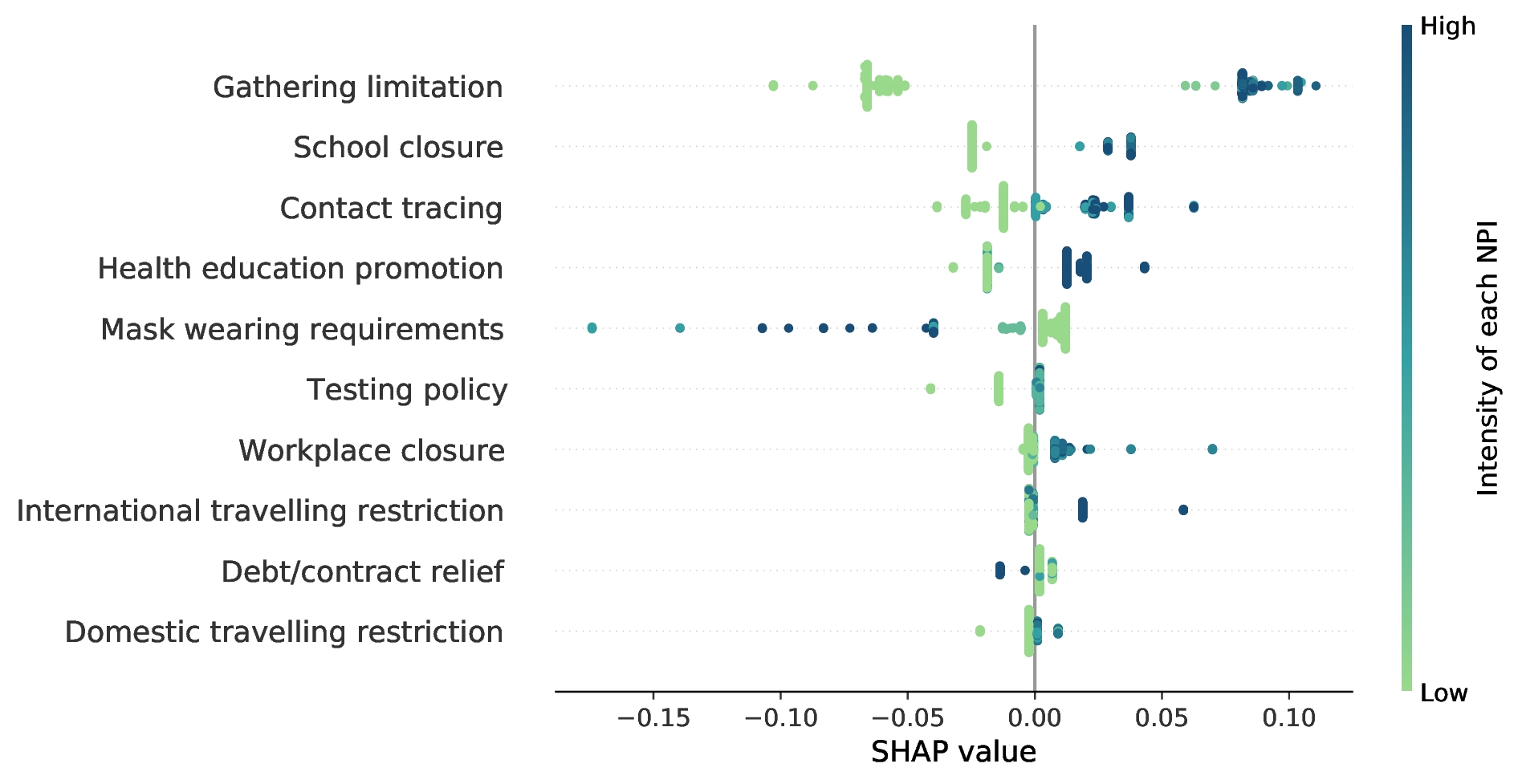


Figure S4: SHAP summary plot: SHAP summary plots for the influenza suppression index show the SHAP values for the 10 NPIs of the respective XGBoost models. The NPIs in the summary plots (y-axis) were ranked by their mean absolute SHAP values, which represent the importance of the NPIs in determining the prediction of the model. The horizontal position (x-axis) of a dot represents the effect of a specific intensity of an NPI on the influenza suppression index for a country at a given point in time.


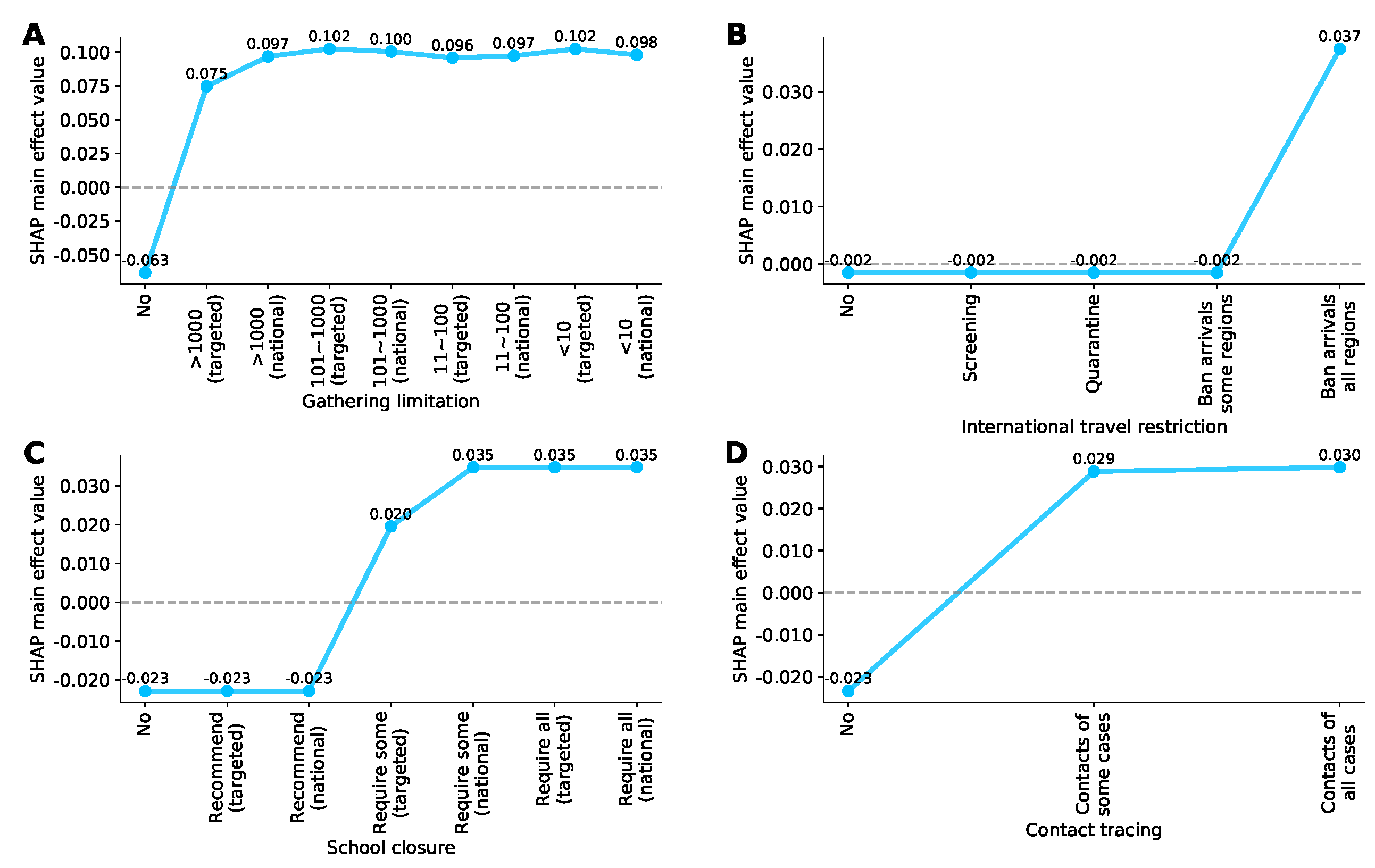

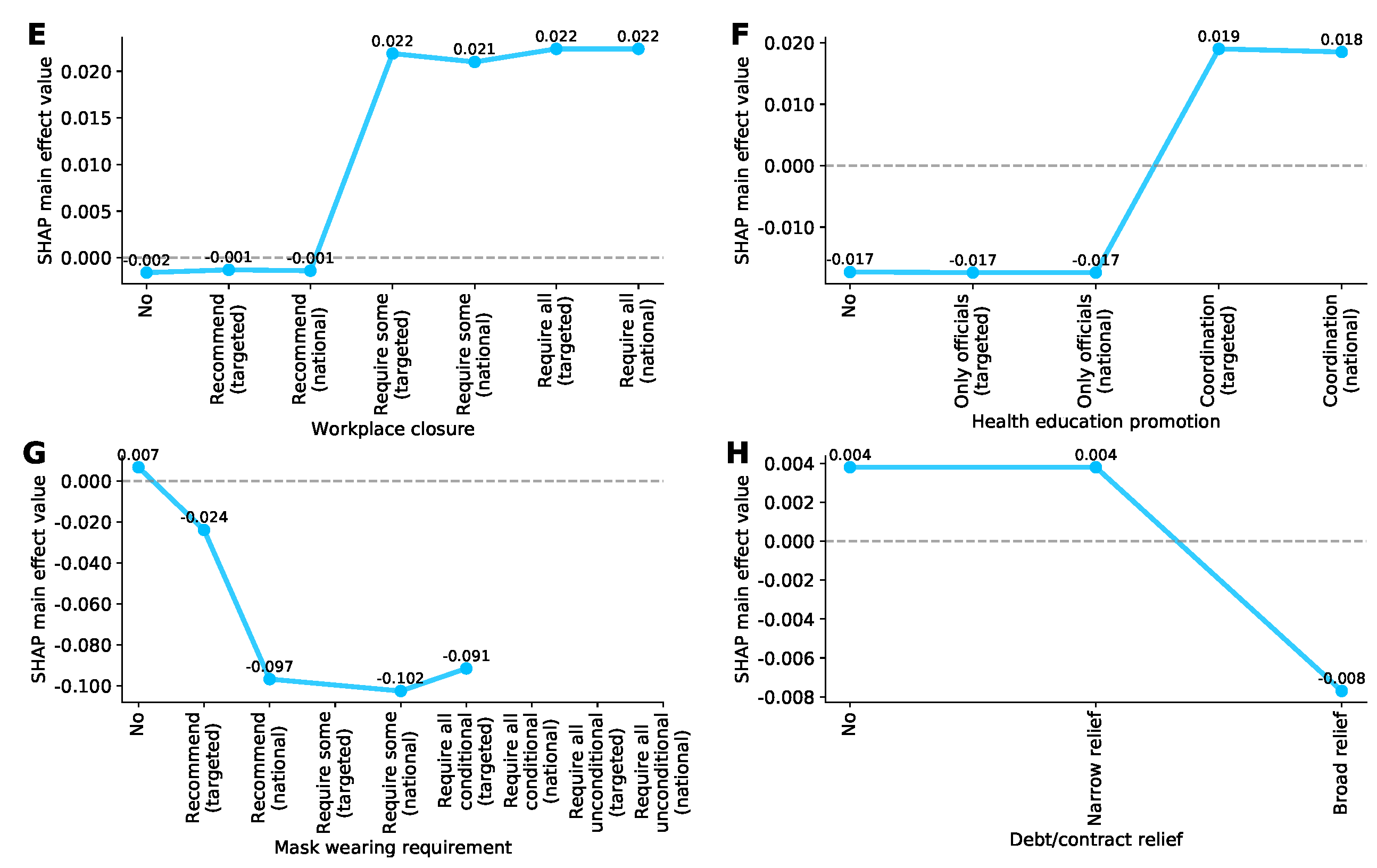

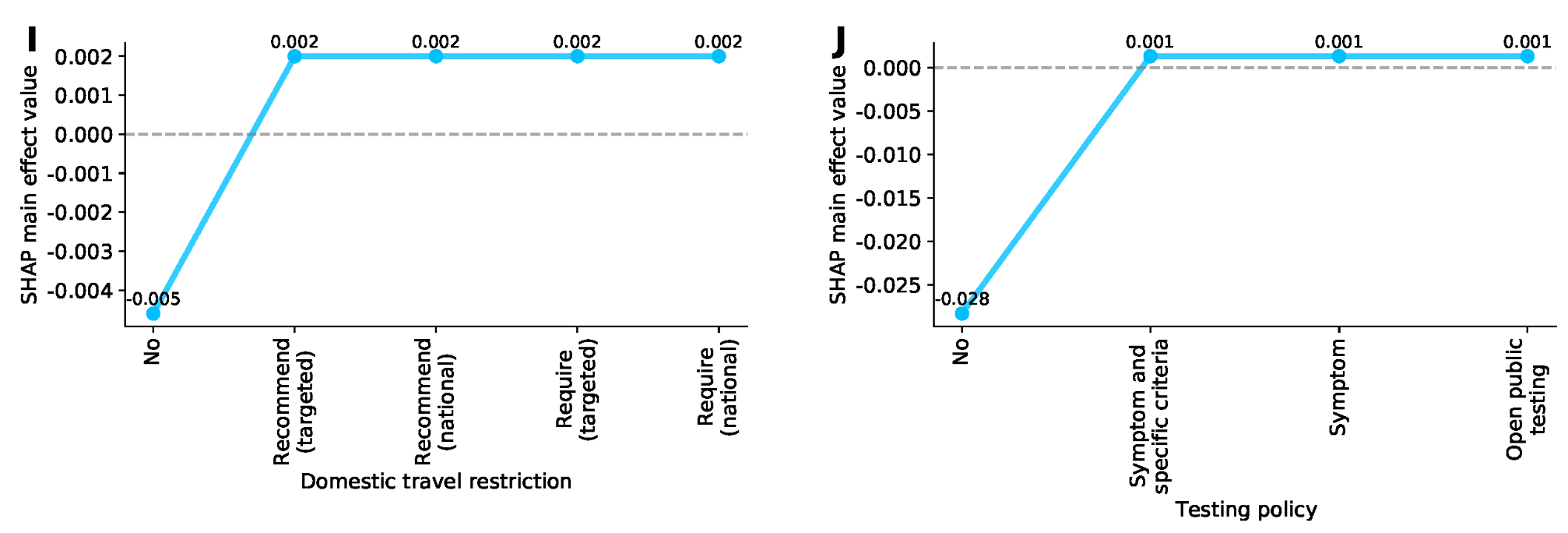


Figure S5: SHAP main effect value of each NPI at different intensity levels. For each NPI, the first time that its SHAP main effect value exceeded zero represents that the NPI with the corresponding intensity level started to suppress influenza transmission, and the intensity below this level means that the NPI did not produce effect. The inflection point in the plot corresponds to the intensity of the NPI that could approximately reach the maximal effect.


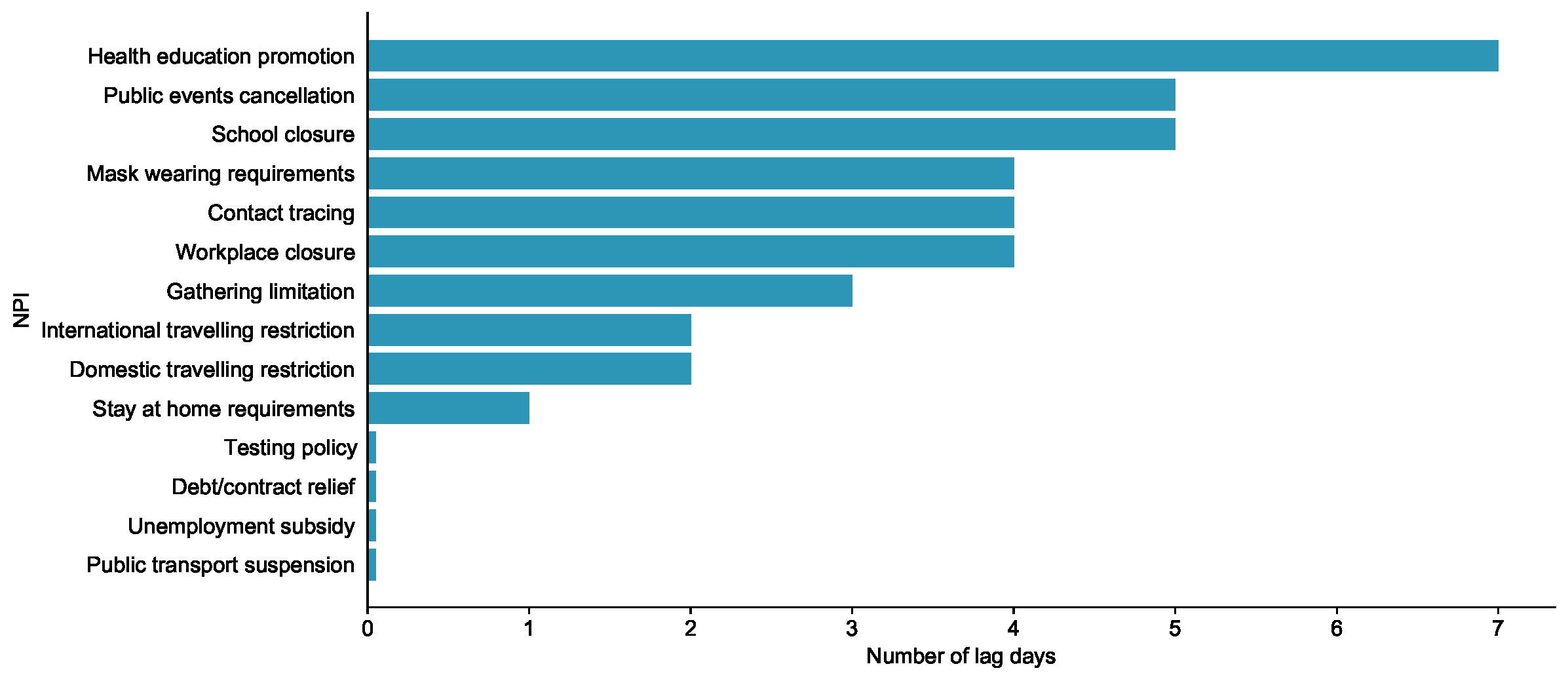


# Figure S6: The time need to take effect for each NPI


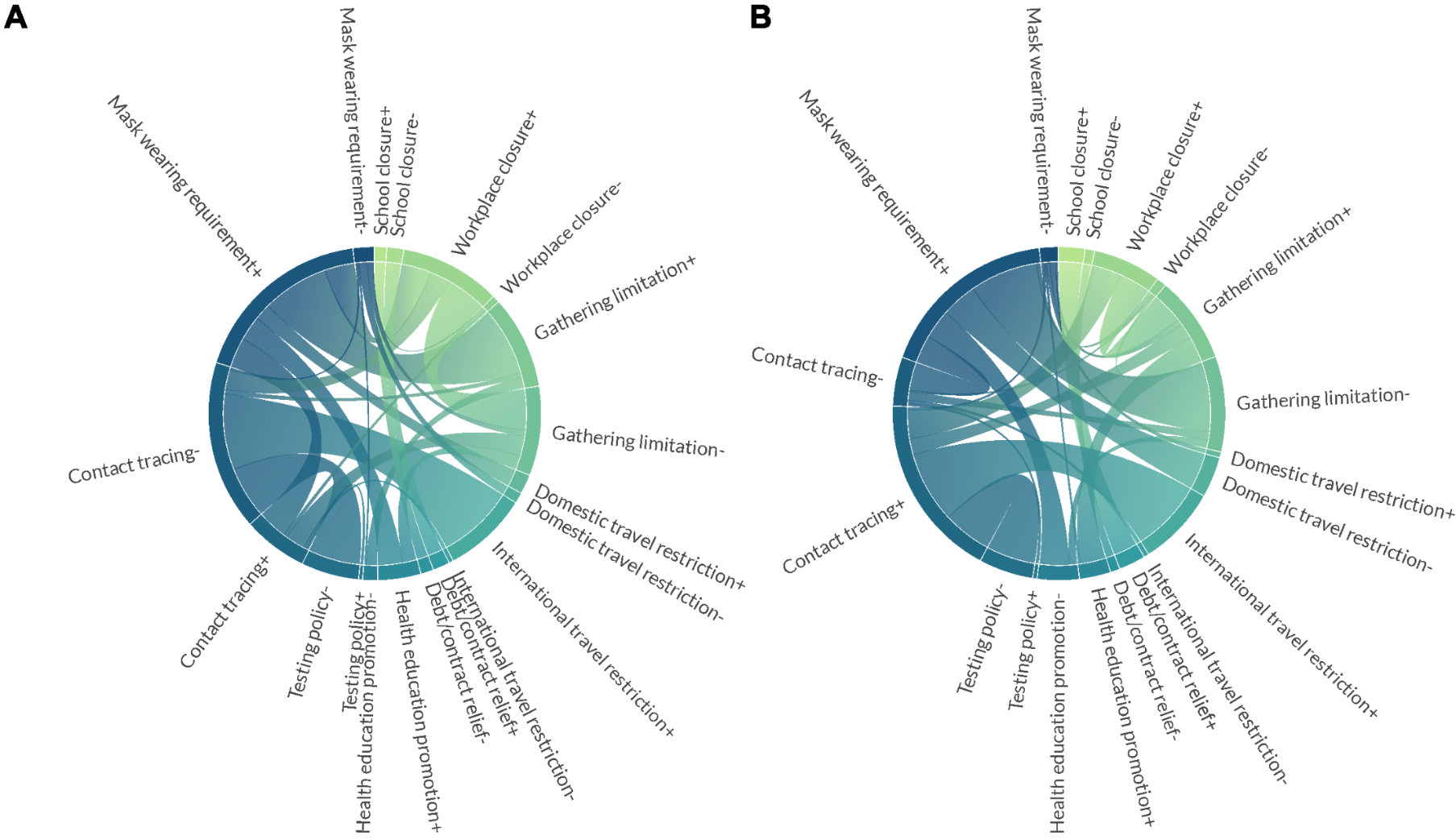


Figure S7: Interaction between each pair of NPIs. (A) Positive interaction; (B) Negative interaction; The plus sign ($+$) after the name of NPI denotes that this NPI exceeds its minimal intensity to take effect; The minus sign ($-$) after the name of NPI denotes that this NPI is below its minimal intensity to take effect; The width of the band between each pair of NPIs denotes the size of SHAP interaction value. Different colors represent different NPIs.


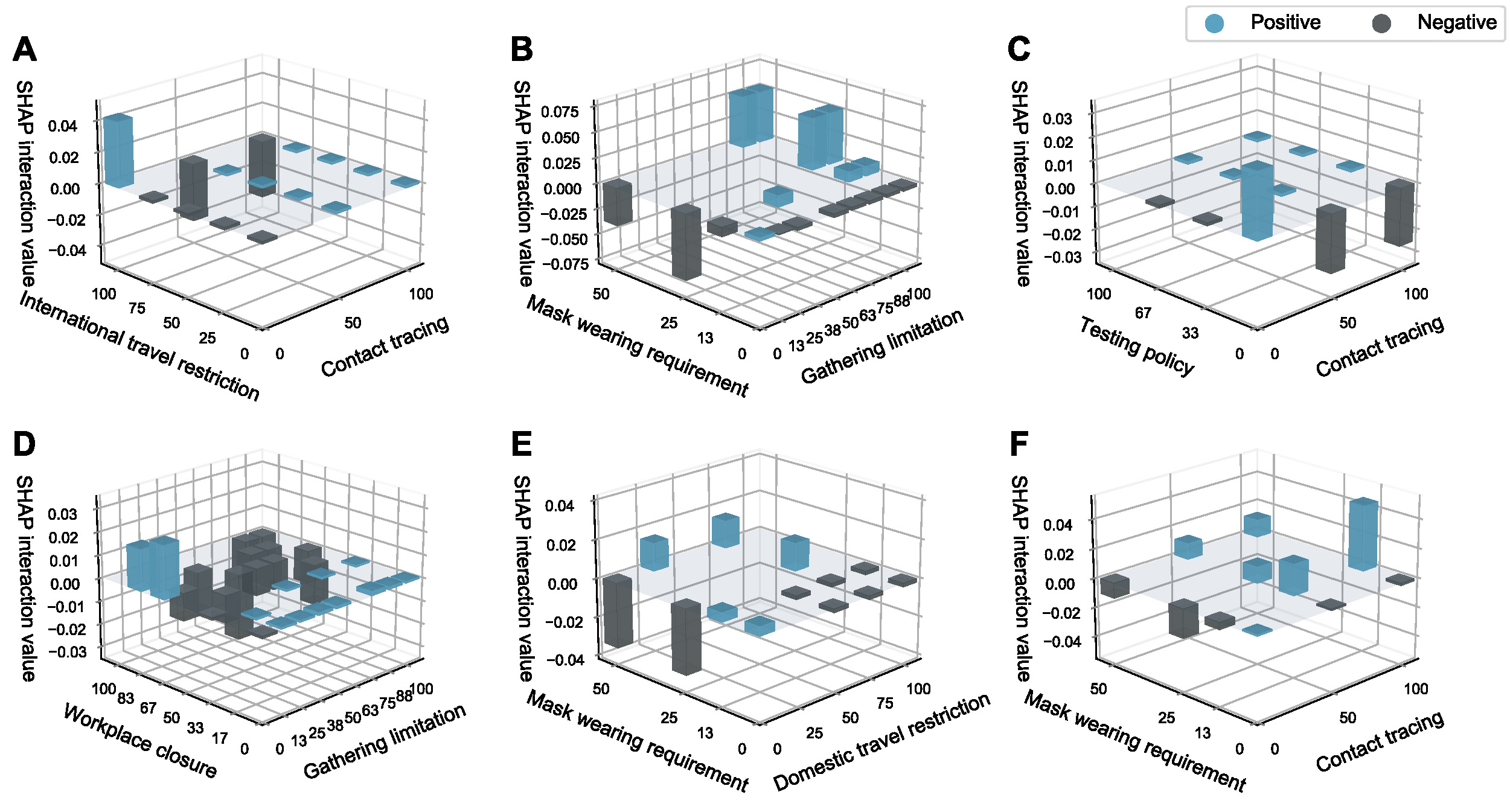

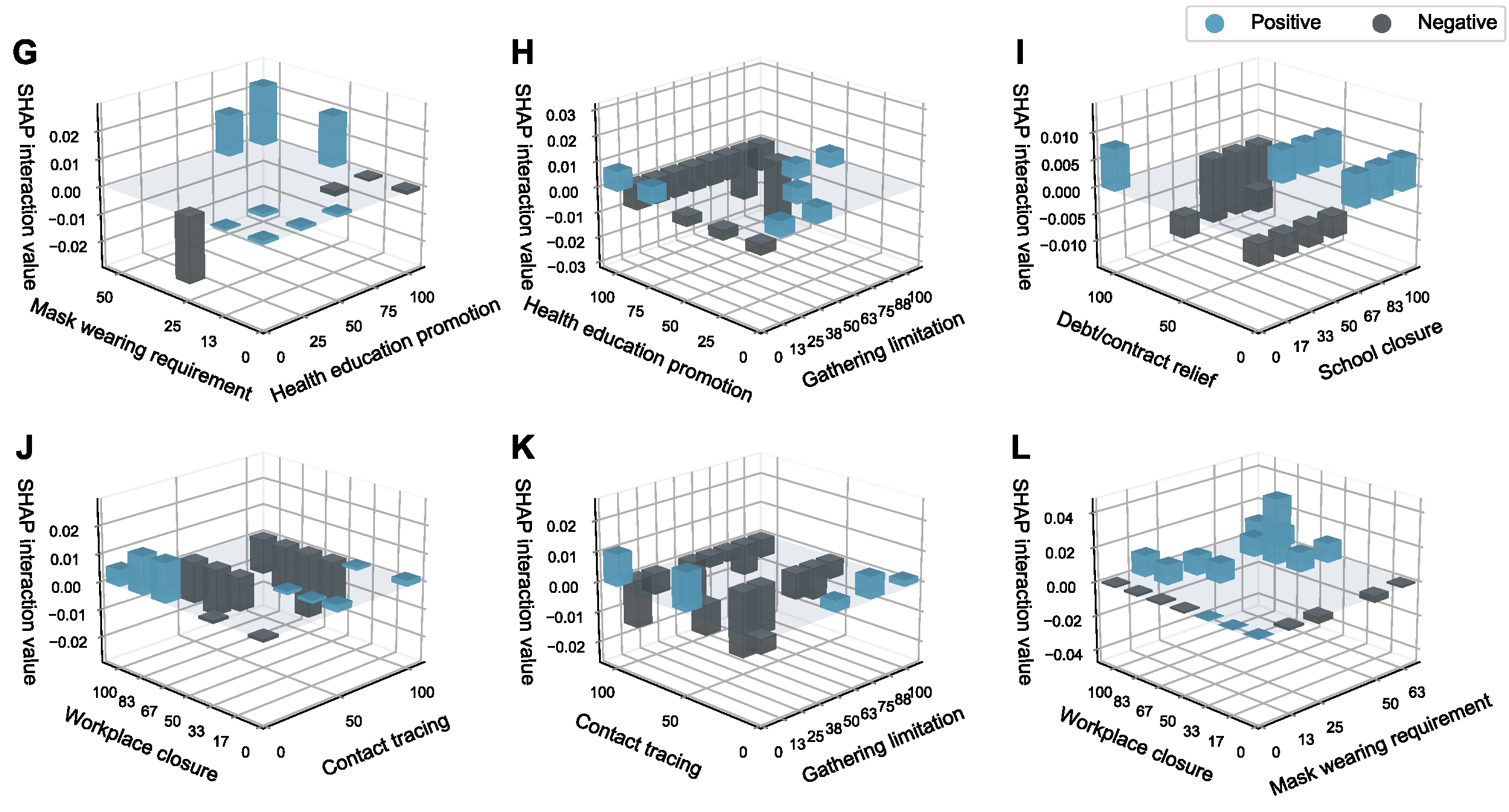


# Figure S8: SHAP interaction value of pair of NPIs


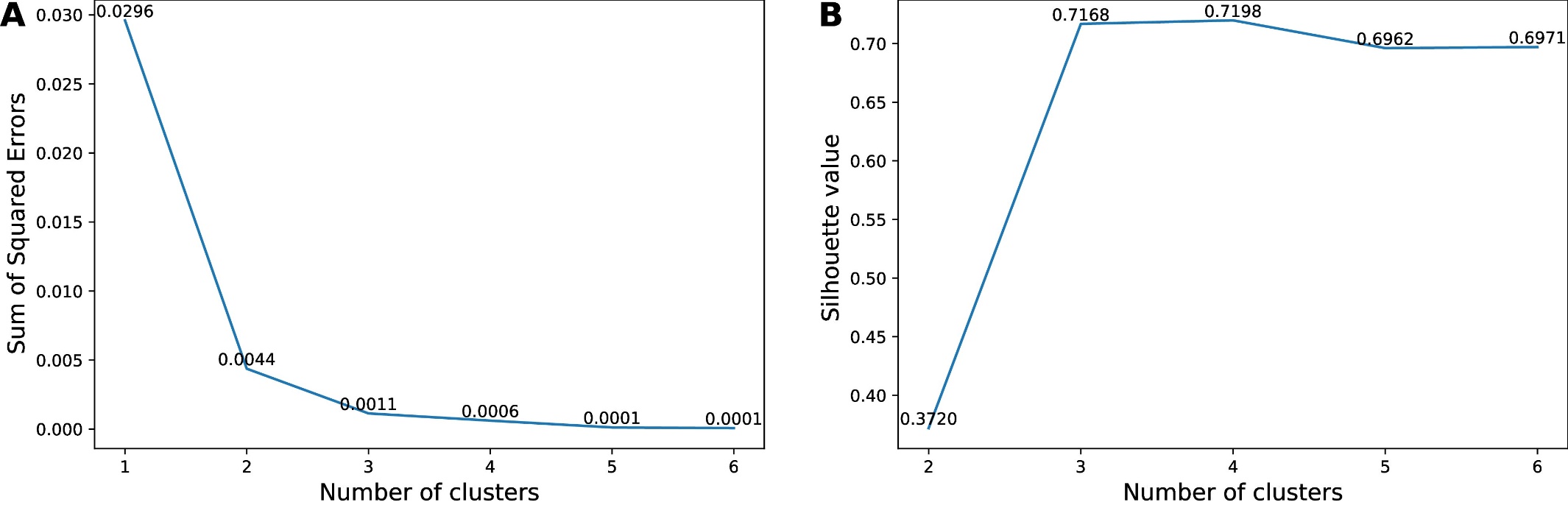


# Figure S9 Sum of squared errors curves. A) Elbow method for an optimal number of initial centroids. B) Silhouette method for an optimal number of initial centroids.

Table S1 The description of each NPI(10)

| NPI | Implementation level and corresponding description | Implementation scope^a^ | Intensity value |
| --- | --- | --- | --- |
| School closure | (0) no measures |  | 0 |
|  | (1) recommend closing or all schools open with alterations resulting in significant differences compared to non-Covid-19 operations | 0 | 17 |
|  |  | 1 | 33 |
|  | (2) require closing (only some levels or categories, e.g., just high school, or just public schools) | 0 | 50 |
|  |  | 1 | 67 |
|  | (3) require closing all levels | 0 | 83 |
|  |  | 1 | 100 |
| Workplace closure | (0) no measures |  | 0 |
|  | (1) recommend closing (or recommend work from home) | 0 | 17 |
|  |  | 1 | 33 |
|  | (2) require closing (or work from home) for some sectors or categories of workers | 0 | 50 |
|  |  | 1 | 67 |
|  | (3) require closing (or work from home) for all-but-essential workplaces (e.g., grocery stores, doctors) | 0 | 83 |
|  |  | 1 | 100 |
| Public events cancellation | (0) no measures |  | 0 |
|  | (1) recommend cancelling | 0 | 25 |
|  |  | 1 | 50 |
|  | (2) require cancelling | 0 | 75 |
|  |  | 1 | 100 |
| Gathering limitation | (0) no measures |  | 0 |
|  | (1) restrictions on very large gatherings (the limit is above 1000 people) | 0 | 13 |
|  |  | 1 | 25 |
|  | (2) restrictions on gatherings between 101-1000 people | 0 | 38 |
|  |  | 1 | 50 |
|  | (3) restrictions on gatherings between 11-100 people | 0 | 63 |
|  |  | 1 | 75 |
|  | (4) restrictions on gatherings of 10 people or less | 0 | 88 |
|  |  | 1 | 100 |
| Public transport suspension | (0) no measures |  | 0 |
|  | (1) recommend closing (or significantly reduce volume/route/means of transport available) | 0 | 25 |
|  |  | 1 | 50 |
|  | (2) require closing (or prohibit most citizens from using it) | 0 | 75 |
|  |  | 1 | 100 |
| Stay at home requirement | (0) no measures |  | 0 |
|  | (1) recommend not leaving house | 0 | 17 |
|  |  | 1 | 33 |
|  | (2) require not leaving house with exceptions for daily exercise, grocery shopping, and 'essential' trips | 0 | 50 |
|  |  | 1 | 67 |
|  | (3) require not leaving house with minimal exceptions (e.g., allowed to leave once a week, or only one person can leave at a time, etc.) | 0 | 83 |
|  |  | 1 | 100 |
| Domestic travel restriction | (0) no measures |  | 0 |
|  | (1) recommend not to travel between regions/cities | 0 | 25 |
|  |  | 1 | 50 |
|  | (2) internal movement restrictions in place | 0 | 75 |
|  |  | 1 | 100 |
| International travel restriction | (0) no measures |  | 0 |
|  | (1) screening arrivals |  | 25 |
|  | (2) quarantine arrivals from some or all regions |  | 50 |
|  | (3) ban arrivals from some regions |  | 75 |
|  | (4) ban on all regions or total border closure |  | 100 |
| Unemployment subsidy | (0) no measures |  | 0 |
|  | (1) government is replacing less than 50% of lost salary (or if a flat sum, it is less than 50% median salary) | 0 | 25 |
|  |  | 1 | 50 |
|  | (2) government is replacing 50% or more of lost salary (or if a flat sum, it is greater than 50% median salary) | 0 | 75 |
|  |  | 1 | 100 |
| Debt/contract relief | (0) no measures |  | 0 |
|  | (1) narrow relief, specific to one kind of contract |  | 50 |
|  | (2) broad debt/contract relief |  | 100 |
| Health education promotion | (0) no measures |  | 0 |
|  | (1) public officials urging caution about Covid-19 | 0 | 25 |
|  |  | 1 | 50 |
|  | (2) coordinated public information campaign (e.g., across traditional and social media) | 0 | 75 |
|  |  | 1 | 100 |
| Testing policy | (0) no measures |  | 0 |
|  | (1) only those who both (a) have symptoms AND (b) meet specific criteria (e.g., key workers, admitted to hospital, came into contact with a known case, returned from overseas) |  | 33 |
|  | (2) testing of anyone showing Covid-19 symptoms |  | 67 |
|  | (3) open public testing (e.g., "drive through" testing available to asymptomatic people) |  | 100 |
| Contact tracing | (0) no measures |  | 0 |
|  | (1) limited contact tracing; not done for all cases |  | 50 |
|  | (2) comprehensive contact tracing; done for all identified cases |  | 100 |
| Mask wearing requirement | (0) no measures |  | 0 |
|  | (1) Recommended | 0 | 13 |
|  |  | 1 | 25 |
|  | (2) Required in some specified shared/public spaces outside the home with other people present, or some situations when social distancing not possible | 0 | 38 |
|  |  | 1 | 50 |
|  | (3) Required in all shared/public spaces outside the home with other people present or all situations when social distancing not possible | 0 | 63 |
|  |  | 1 | 75 |
|  | (4) Required outside the home at all times regardless of location or presence of other people | 0 | 88 |
|  |  | 1 | 100 |

a: 0=Implemented in target regions; 1=Implemented nationwide

# Table S2 Results of sensitivity analysis

|  | Exclude 1 | Exclude 2 | Unnormalized | Median | Truncated mean | No target | RF+SHAP | Lasso | SVR+SFS | RF+SFS |
| --- | --- | --- | --- | --- | --- | --- | --- | --- | --- | --- |
| Gathering limitation* | $\boldsymbol{\surd}$ | $\surd$ | $\surd$ | $\boldsymbol{\surd}$ | $\boldsymbol{\surd}$ | $\surd$ | $\surd$ | $\surd$ | $\surd$ | $\surd$ |
| School closure* | $\surd$ |  | $\surd$ | $\surd$ | $\surd$ | $\surd$ | $\surd$ | $\surd$ | $\surd$ | $\surd$ |
| Contact tracing* | $\surd$ | $\surd$ | $\surd$ | $\surd$ | $\surd$ |  |  | $\surd$ |  |  |
| Health education promotion | $\surd$ | $\surd$ |  |  |  |  |  | $\surd$ |  | $\surd$ |
| Mask wearing requirement |  |  | $\surd$ | $\surd$ | $\surd$ |  | $\surd$ |  | $\surd$ |  |
| Workplace closure |  |  |  |  |  |  |  |  | $\surd$ |  |
| International travel restriction |  |  |  |  |  |  |  |  | $\surd$ |  |
| Debt/contract relief |  |  |  |  |  |  |  |  |  | $\surd$ |
| Testing policy |  |  |  |  |  |  |  |  | $\surd$ | $\surd$ |
| Domestic travel restriction |  |  |  |  |  |  |  |  |  |  |
| Public events cancellation |  |  |  |  |  |  |  |  | $\surd$ | $\surd$ |
| Public transport suspension |  |  |  |  |  |  |  |  |  |  |
| Stay at home requirement |  |  |  |  |  |  |  |  |  | $\surd$ |
| Unemployment subsidy |  |  |  |  |  |  |  | $\surd$ |  |  |

The order of NPI in the first column is determined by the ranking of contribution in the main result, with the largest contribution in the first row.

*: The contribution of NPIs > 10%.

Exclude1: each country was removed one at a time and the analysis was repeated for a total of 33 times; Exclude2: Canada, China, Russia, and United States were excluded from our main analysis; Unnormalized: the unnormalized influenza-positive rate was used; Median: influenza-positive rate from the previous 9 years were averaged by calculating the median; Truncated mean: influenza-positive rate from the previous 9 years were averaged by calculating the truncated mean (with the maximum and minimum values removed); No target: intensity value was not taken into account; SFS: sequential feature selection; SVR: support vector machine; RF: random forest.

(1) In Exclude1, the NPIs being selected (The contribution of NPIs >10%) for more than 16 times (>50%) were ticked.

(2) The contribution of NPIs >10% in these methods (Exclude2, Unnormalized, Median, Truncated mean, No target, and RF+SHAP) were ticked.

(3) NPIs retained in the final model after feature selection by these methods (Lasso, SVR+SFS, and RF+SFS) were ticked.

# Table S3 One-sample Wilcoxon test for the SHAP main effect value

| **NPI** | **Intensity** | **Wilcoxon_P** |
| --- | --- | --- |
| School closure | 0 | 1.000 |
| School closure | 33.3 | 1.000 |
| School closure | 50 | 0.063 |
| School closure | 66.7 | <0.001* |
| School closure | 83.3 | <0.001* |
| School closure | 100 | <0.001* |
| Workplace closure | 0 | 1.000 |
| Workplace closure | 33.3 | 1.000 |
| Workplace closure | 50 | 0.002* |
| Workplace closure | 66.7 | <0.001* |
| Workplace closure | 83.3 | <0.001* |
| Workplace closure | 100 | <0.001* |
| Gathering limitation | 0 | 1.000 |
| Gathering limitation | 12.5 | 0.016* |
| Gathering limitation | 25 | 0.063 |
| Gathering limitation | 37.5 | 0.250 |
| Gathering limitation | 50 | <0.001* |
| Gathering limitation | 62.5 | 0.250 |
| Gathering limitation | 75 | <0.001* |
| Gathering limitation | 87.5 | <0.001* |
| Gathering limitation | 100 | <0.001* |
| Domestic travel restriction | 0 | 1.000 |
| Domestic travel restriction | 25 | 0.125 |
| Domestic travel restriction | 50 | <0.001* |
| Domestic travel restriction | 75 | <0.001* |
| Domestic travel restriction | 100 | <0.001* |
| International travel restriction | 0 | 1.000 |
| International travel restriction | 25 | 1.000 |
| International travel restriction | 50 | 1.000 |
| International travel restriction | 75 | 1.000 |
| International travel restriction | 100 | <0.001* |
| Debt/contract relief | 0 | <0.001* |
| Debt/contract relief | 50 | <0.001* |
| Debt/contract relief | 100 | 1.000 |
| Health education promotion | 0 | 1.000 |
| Health education promotion | 25 | 1.000 |
| Health education promotion | 50 | 1.000 |
| Health education promotion | 75 | 0.016* |
| Health education promotion | 100 | <0.001* |
| Testing policy | 0 | 1.000 |
| Testing policy | 33.3 | <0.001* |
| Testing policy | 66.7 | <0.001* |
| Testing policy | 100 | <0.001* |
| Contact tracing | 0 | 1.000 |
| Contact tracing | 50 | <0.001* |
| Contact tracing | 100 | <0.001* |
| Mask wearing requirement | 0 | <0.001* |
| Mask wearing requirement | 12.5 | 1.000 |
| Mask wearing requirement | 25 | 1.000 |
| Mask wearing requirement | 50 | 1.000 |
| Mask wearing requirement | 62.5 | 1.000 |

NPI: nonpharmaceutical interventions

Intensity: The implementation intensity value of the NPI

Wilcoxon_P: The P-value of one-sample Wilcoxon signed-rank test.

*: P-value lower than 0.05

# References

1. Chen. T, Guestrin C. XGBoost: A Scalable Tree Boosting System. In 22nd SIGKDD Conference on Knowledge Discovery and Data Mining. 2016.

2. Lundberg SM, Erion G, Chen H, DeGrave A, Prutkin JM, Nair B, et al. From Local Explanations to Global Understanding with Explainable AI for Trees. Nat Mach Intell. 2020;2(1):56-67.

3. Friedman J, Hastie T, Tibshirani R. Regularization Paths for Generalized Linear Models via Coordinate Descent. J Stat Softw. 2010;33(1).

4. Breiman L. Random Forests. Machine Learning. 2001;45(1):5-32.

5. Platt J. Probabilistic outputs for support vector machines and comparison to regularizedlikelihood methods. Advances in Large Margin Classifiers. 1999.

6. Ferri FJ PP, Hatef M, Kittler J. Comparative study of techniques for large-scale feature selection. Machine Intelligence and Pattern Recognition. 1994.

7. Qualls M, Pallin DJ, Schuur JD. Parametric versus nonparametric statistical tests: the length of stay example. Acad Emerg Med. 2010;17(10):1113-21.

8. Mahendru K. How to Determine the Optimal K for K-Means? 2017 [Available from: https://medium.com/analytics-vidhya/how-to-determine-the-optimal-k-for-k-means-708505d204eb.

9. Alade T. Tutorial: How to determine the optimal number of clusters for k-means clustering. 2018.

10. Hale T, Angrist N, Goldszmidt R, Kira B, Petherick A, Phillips T, et al. A global panel database of pandemic policies (Oxford COVID-19 Government Response Tracker). Nat Hum Behav. 2021;5(4):529-38.
